# The mitochondrial citrate carrier, SLC25A1, drives stemness and therapy resistance in non-small cell lung cancer

**DOI:** 10.1038/s41418-018-0101-z

**Published:** 2018-04-12

**Authors:** Harvey R. Fernandez, Shreyas M. Gadre, Mingjun Tan, Garrett T. Graham, Rami Mosaoa, Martin S. Ongkeko, Kyu Ah Kim, Rebecca B. Riggins, Erika Parasido, Iacopo Petrini, Simone Pacini, Amrita Cheema, Rency Varghese, Habtom W Ressom, Yuwen Zhang, Christopher Albanese, Aykut Üren, Mikell Paige, Giuseppe Giaccone, Maria Laura Avantaggiati

**Affiliations:** 1 Georgetown University Medical Center, Lombardi Comprehensive Cancer Center, Washington D.C, 20057 USA; 2 Chemistry and Biochemistry Department, George Mason University, Fairfax, VA USA; 3 Department of Clinical and Experimental Medicine, Department of Surgical, Medical and Molecular Pathology and Critical Care Medicine University of Pisa, Pisa, Italy

## Abstract

Therapy resistance represents a clinical challenge for advanced non-small cell lung cancer (NSCLC), which still remains an incurable disease. There is growing evidence that cancer-initiating or cancer stem cells (CSCs) provide a reservoir of slow-growing dormant populations of cells with tumor-initiating and unlimited self-renewal ability that are left behind by conventional therapies reigniting post-therapy relapse and metastatic dissemination. The metabolic pathways required for the expansion of CSCs are incompletely defined, but their understanding will likely open new therapeutic opportunities. We show here that lung CSCs rely upon oxidative phosphorylation for energy production and survival through the activity of the mitochondrial citrate transporter, SLC25A1. We demonstrate that SLC25A1 plays a key role in maintaining the mitochondrial pool of citrate and redox balance in CSCs, whereas its inhibition leads to reactive oxygen species build-up thereby inhibiting the self-renewal capability of CSCs. Moreover, in different patient-derived tumors, resistance to cisplatin or to epidermal growth factor receptor (EGFR) inhibitor treatment is acquired through SLC25A1-mediated implementation of mitochondrial activity and induction of a stemness phenotype. Hence, a newly identified specific SLC25A1 inhibitor is synthetic lethal with cisplatin or with EGFR inhibitor co-treatment and restores antitumor responses to these agents in vitro and in animal models. These data have potential clinical implications in that they unravel a metabolic vulnerability of drug-resistant lung CSCs, identify a novel SLC25A1 inhibitor and, lastly, provide the first line of evidence that drugs, which block SLC25A1 activity, when employed in combination with selected conventional antitumor agents, lead to a therapeutic benefit.

## Introduction

Non-small cell lung cancer (NSCLC) causes thousands of deaths annually in the United States. Treatment of NSCLC has undergone significant changes recently [1–3]. Targeted therapies against various driver mutations including the epidermal growth factor receptor (EGFR) have improved outcome in NSCLC patients whose tumors harbor these genetic abnormalities, whereas platinum-based chemotherapy remains the treatment of choice for most patients with tumors without “druggable” targets [3–5]. The principal cause of mortality in NSCLC is the development of drug resistance and metastatic disease. Although intra-tumoral genetic heterogeneity is a key contributor to resistance, tumor cells exhibit phenotypic plasticity that allows them to alter their growth characteristics enabling adaptation to the tumor microenvironment, as well as to therapeutic attacks [6–8]. Cells with a stem-like, dormant phenotype, endowed with unlimited self-renewal and high tumorigenic capability, are deemed responsible for post-therapy relapse and metastatic dissemination in various cancers, including lung cancer [9–14]. This has led to the proposal that drugs that attack the cancer stem cell (CSC) population have therapeutic benefit.

The understanding of the metabolic pathways required by tumor cells is now seen as a critical component for the development of tumor therapeutics [15]. While in the past, many studies focused on the glycolytic behavior of “bulk” highly proliferating cells, recent literature has highlighted the requirement for mitochondrial respiration in metastatic breast and pancreatic cancer and in Li–Fraumeni syndrome [16–20]. Importantly, tumor cells are not only genetically, but also metabolically heterogeneous being able to utilize different metabolic pathways depending upon proliferation rates and also based upon their intra-tumoral “geographical location”. Cancer stem-like cells that are resistant to therapy survive for long periods of time in a dormant state, residing in niches deprived of oxygen and nutrients, an environment restrictive for the growth of highly proliferating cells [21, 22]. This slow-growing dormant state is proposed to allow CSCs to tolerate anti-proliferative signals conveyed by therapeutic attacks protecting them from pro-death stimuli. Moreover, although the energetic output of glycolysis is inferior compared with oxidative phosphorylation, glycolysis is advantageous for highly proliferating cells that need to derive energy at fast rates, whereas quiescent cells do not utilize this pathway as the preferential energy source [22, 23]. Thus, the metabolic requirements of CSCs most probably differ from those of cells with highly proliferative capacity.

In this study, we focus our attention on SLC25A1, a mitochondrial carrier that promotes the flux of citrate/isocitrate across the mitochondria, in exchange for the entry of cytosolic malate [24, 25]. Although in the cytoplasm citrate is the precursor for lipogenesis, in the mitochondria it enters the Krebs cycle promoting mitochondrial respiration. Previously, we proposed that SLC25A1 is a metabolic oncogene [26, 27], but its importance in cancer therapy is still unknown. Here, we characterize novel activities of SLC25A1 in the stem cell population and we identify a new SLC25A1 inhibitor compound with promising activity in drug-resistant tumors.

## Results

### SLC25A1 promotes self-renewal of CSCs

**Fig. 1:**
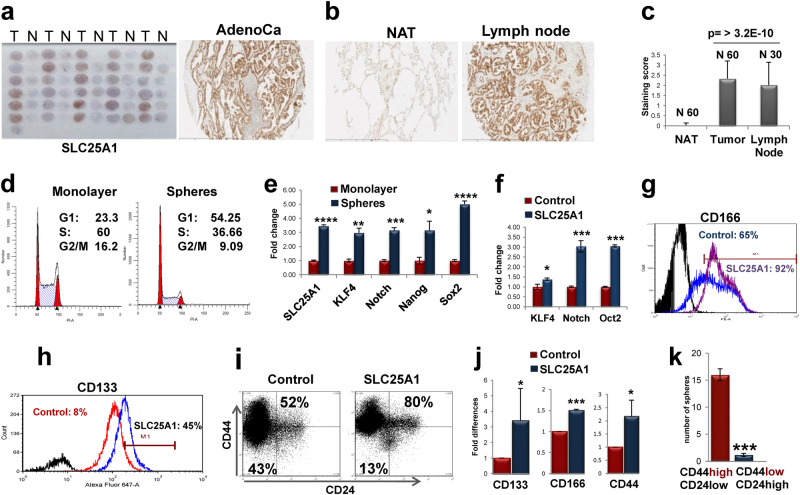
SLC25A1 expression correlates with metastatic disease and promotes stemness in NSCLC. **a** Representative SLC25A1 immunohistochemical staining of tissue microarrays of lung adenocarcinomas. **b** Representative images of an immunoreactive tumor, normal adjacent tissue (NAT) and metastatic lymph node. **c** Quantitative analysis of SLC25A1 staining based on percentage of reactive cells. **d** The cell cycle profile of H1299 cells grown as monolayers or spheres is shown. **e** Quantitative real-time PCR (normalized values) on the indicated gene products performed in either monolayer (red bars) or spheres of H1299 cells and analyzed in triplicate samples. **f** Real-time PCR of the indicated genes performed in naive H1299 or SLC25A1-expressing cells. **g**–**i** FACS analysis of CD166, CD133 expression and of the CD44 high, CD24 low population in naive H1299 and SLC25A1-expressing cells. **j** Quantification of the indicated markers from three independent experiments each with duplicate values. **k** To study self-renewal, cells from SLC25A1-spheres were sorted for CD44/CD24 high or low populations and re-plated independently as single cells.

To elucidate the relevance of SLC25A1 in NSCLC, we performed immunohistochemical analysis of tissue microarrays containing 90 NSCLCs, as well as matched normal adjacent tissues (NATs) and metastatic lymph nodes. Nearly all adenocarcinomas (Figs. 1a–c) and squamous carcinomas (Supplementary Fig.1a-c) were immunoreactive for SLC25A1, unlike the normal respiratory epithelium. Importantly, the metastatic foci in lymph nodes were all positive for SLC25A1 (Figs. 1b, c), demonstrating that SLC25A1 is highly expressed at metastatic sites.

**Fig. 2:**
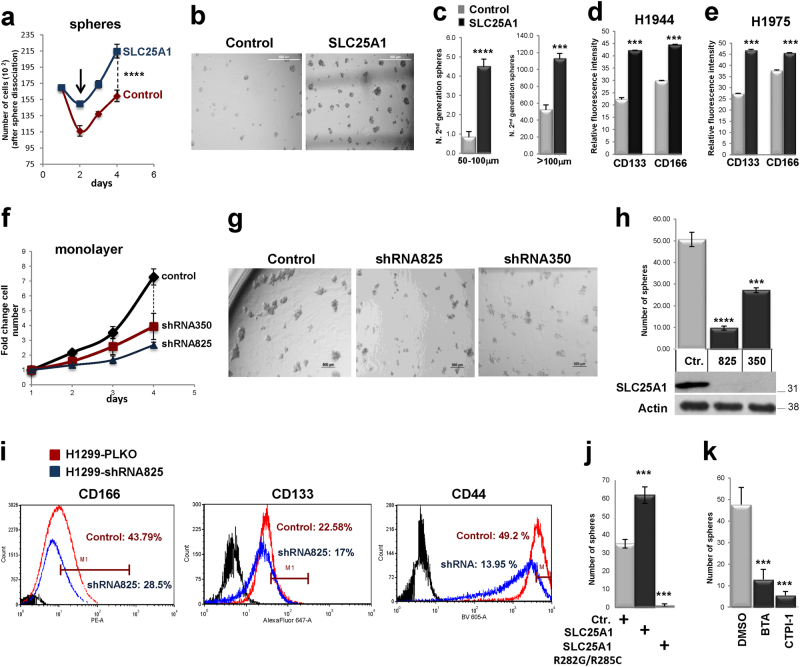
**a** Growth curves of H1299 or SLC25A1-expressing cells cultured as spheres, dissociated and counted at each of the indicated time points. The arrows indicate the loss of viability that occurs in the first days of sphere formation, likely reflective of differentiated cells dying by anoikis. **b** Control or SLC25A1-expressing cells were dissociated and plated as single spheres in semisolid media, methylcellulose. After 1 week, spheres were isolated, dissociated again and re-plated in the same conditions, producing second-generation spheres. **c** Quantification of the experiments shown in **b.** **d**, **e** FACS analysis of CD133 and CD166 in the indicated cell lines either mock transfected (gray bars) or transfected with the vector expressing SLC25A1 (black bars). **f** Growth curves of H1299 cells infected with lentivirus control shRNA, or with either of two SLC25A1 shRNAs (825 and 350). **g**,** h** Sphere-forming ability of H1299 cells infected with lentivirus control or with the indicated SLC25A1-shRNAs, dissociated and plated as single cells in semisolid media. **i** CD166 and CD44 expression in cells infected with PLKO control lentivirus or with lentivirus harboring the specific SLC25A1 shRNA. **j**,** k** Sphere-forming ability of cells expressing SLC25A1 wild-type or mutant SLC25A1R282G/R285C **j**, or treated with the SLC25A1 inhibitors BTA (2 mM) and CTPI (1 mM) **k**. Bars represent the standard deviation, asterisks refer to *P ≤ 0.05; **P ≤ 0.01; ***P ≤ 0.001; ****P ≤ 0.0001 by unpaired *T*-test

We previously reported that SLC25A1-expressing cells are highly tumorigenic when injected in nude mice [26]. As CSCs are the source of cancer initiation, we asked whether SLC25A1 affects the expansion of this population. We therefore employed anchorage-independent spheroid assays grown in defined growth factor media and without serum (which promotes differentiation). CSCs are able to expand and are enriched in these conditions, whereas differentiated cells undergo anoikis [28, 29]. Self-renewal was assessed by monitoring the growth of first- and second-generation spheres and by plating cells in semisolid media, which prevents cell-to-cell contacts and, thus, only allows growth of monoclonal populations [29]. Spheres derived from the lung adenocarcinoma cell line, H1299, exhibited a contraction of the S-phase of the cell cycle compared with monolayer cultures (Fig. 1d), consistent with a non-replicating state, and enrichment of the stemness markers KLF4, Notch-1, Nanog and Sox2 (Fig. 1e). Noticeably, SLC25A1 was enriched at both mRNA (Fig. 1e) and protein level in spheres, demonstrating its co-expression with stem cell markers, whereas mitochondrial amount was unaffected (Supplementary Figure 2a). SLC25A1 spheres also overexpressed Notch-1, Oct2, as well as CD133, CD166 and the CD44^high^/CD24^low^ population, which possesses self-renewal capability [13] (Figs. 1f–j). Accordingly, only CD44^high^/CD24^low^, but not CD44^low^/CD24^high^ cells derived from SLC25A1 spheres were able to self-renew and to produce second-generation spheres (Fig. 1k). Further, SLC25A1 promoted the expansion of CSCs (Fig. 2a) and self-renewal on second (Figs. 2b, c) and third passage (data not shown) relative to control cells. To rule out cell type-specific effects, we expanded this analysis to other NSCLC cell lines. Overexpression of SLC25A1 in H1975 or in H1944 cells similarly enhanced CD133 and CD166 expression (Figs. 2d, e).

We then assessed the effects of the SLC25A1 knock-down with two short hairpin RNAs (shRNAs). In spite of reduced proliferation rates, cells lacking SLC25A1 continued to grow and could be propagated indefinitely as two-dimensional cultures (Fig. 2f). However, their sphere-forming capacity was severely compromised (Figs. 2g, h, Supplementary Figure 3) and the levels of stem cell markers were reduced (Fig. 2i). Similarly, cells harboring a SLC25A1 mutant where two residues necessary for citrate binding, Arg282 and Arg285, were replaced with glycine or cysteine (SLC25A1R282G/R285C), were unable to form spheres (Fig. 2j). Finally, treatment with two previously validated SLC25A1 inhibitors [27, 30], benzenetricarboxylate (BTA) and citrate transporter inhibitor-1 (CTPI-1), also reduced sphere-forming ability (Fig. 2k). These data indicate that SLC25A1 promotes the expansion and self-renewal of lung CSCs and highlight the importance of modeling CSC proliferation in three-dimensional spheroid systems.

### Discovery of a novel SLC25A1 inhibitor compound

**Fig. 3:**
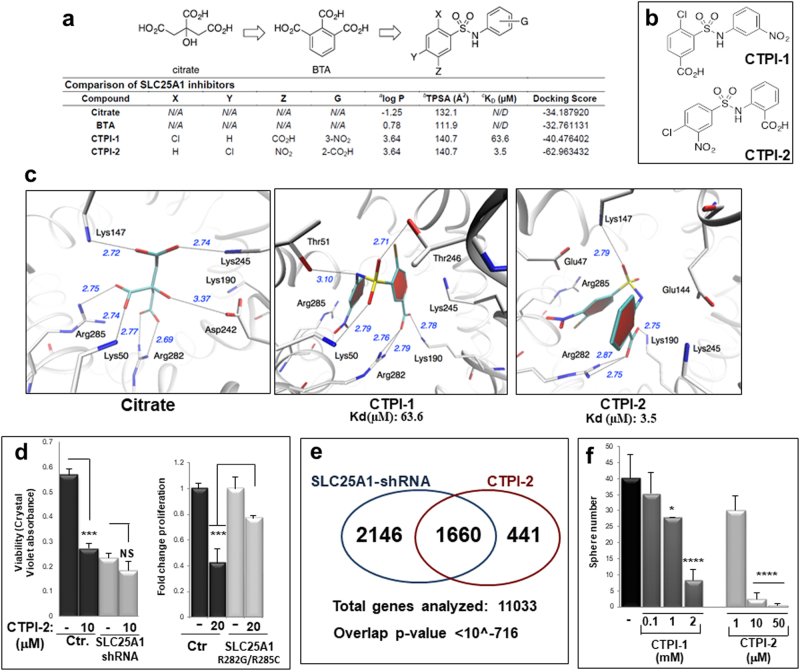
Identification and activity of a new SLC25A1 inhibitor. **a** Comparison of the structure and properties of citrate, BTA, CTPI-1 and CTPI-2 and general structure SLC25A1 inhibitors (top panel): *a*: Calculated octanol–water partition coefficient; *b*: Topical polar surface area (TPSA); *c*: Experimental dissociation constant. *d*: Docking score calculated by the UCSF DOCK6.7 software. **b** The structure of the leading compound (CTPI-1) and of the newly identified SLC25A1 inhibitor (CTPI-2) are shown. **c** A binding model for citrate, CTPI-1 or CTPI-2 in complex with a homology model of *human* SLC25A1. The Dock6.7 software provided a score for binding and a potential pose for the structure. Refinement of the structure using the AMBER MD module in the UCSF Dock6.7 suite of software was performed to give the optimized models. The model shows the relevant amino acids previously known to be involved in citrate binding, including Lys147, Lys245, Arg285, Lys50 and Arg282. **d** Proliferation rates assessed with crystal violet of H1299 transduced with control or with the SLC25A1-shRNA lentivirus or fold proliferation changes of cells transfected with the SLC25A1R282G/R285C and treated with CTPI-2, respectively. **e** Gene expression arrays performed on Illumina Human HT-12 v4 Bead Chip of cells expressing the SLC25A1-shRNA or treated with CTPI-2. A cutoff of fold change >2-fold over control, was used to evaluate the similarity of gene expression profile changes between the shRNA and CTPI-2 treatment. The overlap *p-*value (hypergeometric test) of <10^−716^ is shown. **f** Comparison of the activity of CTPI-1 and CTPI-2 in sphere-forming assays at the indicated concentrations. Asterisks indicate *p*-values calculated from cells plated in five or six independent wells and comparisons refer to untreated control cells

The above findings suggest that SLC25A1 inhibitors may target the CSC population. We previously characterized two SLC25A1 inhibitors, which, owing to a negative partition coefficient and topical polar surface area, do not easily cross the cell membrane [26, 27]. A second inhibitor, CTPI-1, was identified in *yeast* [30]. However, surface plasmon resonance (SPR) spectroscopy experiments revealed that CTPI-1 exhibits suboptimal binding for SLC25A1 (*K*_D_ 63.6 μM, Fig. 3a). One key difference between *yeast* and *human* SLC25A1 is Arg181 in the *yeast* homolog, which is replaced by Lys190 in the *human* protein [30]. To optimize compounds specific for *human* SLC25A1, an *in silico* homology model was derived and docking experiments were performed. By exchanging the Z group to a nitro substituent with the chlorine atom for the Y group of CTPI-1, we identified a compound (CTPI-2) that exhibits an experimental *K*_D_ of 3.5 µM, thus a nearly 20-fold improvement of binding activity relative to CTPI-1 (Figs. 3a–c; Supplementary Figure 2bc). CTPI-1 interacts with SLC25A1 through a network of hydrogen bonds involving amino acids well-known to contribute to citrate binding, specifically Lys245, Lys190, Arg282 and Arg285, allowing for a closed conformation, which takes advantage of intramolecular pi–pi stacking of the two aromatic moieties (Fig. 3c). In comparison, CTPI-2 affords a closer binding mode with Arg282 and Arg285, and the sulfonamide moiety now also aligns toward Lys190. Together, these improved interactions might contribute to the enhancement of CTPI-2 binding to SLC25A1.

We next sought to confirm the specificity of CTPI-2. First, CTPI-2 failed to inhibit proliferation in cells lacking SLC25A1 or expressing the SLC25A1R282G/R285C mutant (Fig. 3d). Second, a closely related scaffold compound of CTPI-2, lacking the functional groups necessary for SLC25A1 binding, did not interact with SLC25A1 (data not shown) and did not affect proliferation rates (Supplementary Figure 2de). Third, the comparison of the gene expression profiles of cells expressing the SLC25A1-shRNA or treated with CTPI-2, demonstrated clearly that approximately 80% of genes detected with CTPI-2 treatment were also found in SLC25A1-shRNA-expressing cells, with a highly significant overlap *p*-value (Fig. 3e). Unsurprisingly, more genes were regulated by the shRNA compared with CTPI-2, likely underlying the existence of citrate-independent SLC25A1 activities. Furthermore, CTPI-2 inhibited sphere-forming capacity more efficiently than CTPI-1 (Fig. 3f). Thus, CTPI-2 treatment largely phenocopies SLC25A1 deficiency, suggesting that SLC25A1 is its principal intracellular target. In summary, we have identified the most potent SLC25A1 inhibitor showing significant improvement in the in vitro binding affinity and in vivo activity.

### SLC25A1 promotes mitochondrial respiratory activity and the invasive ability of lung CSCs

**Fig. 4:**
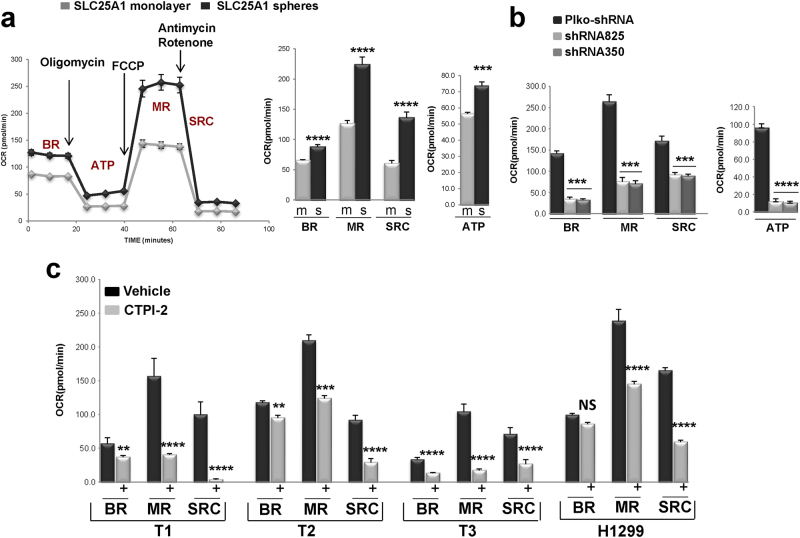
SLC25A1 affects mitochondrial activity and self-renewal in CSCs. **a** Oxygen consumption rate (OCR) were assessed using the Seahorse Extracellular Flux Analyzer after injection of oligomycin (O), FCCP (F) and Antimycin/Rotenone (A + R), in H1299 cells expressing SLC25A1 grown as monolayer or spheres. OCR was normalized to cell number. To eliminate the influence of the media composition and of attachment, for all these experiments monolayer and sphere cultures were first grown for several days, and subsequently they were dissociated, plated on 96-wells coated with the geltrex ECM and pre-equilibrated in the same media containing serum and 5% glucose for 6–12 h, prior to the assessment of the OCR. BR, MR, SRC refer to basal, maximal respiration and spare respiratory capacity. **b** OCR rates in control or in cells expressing SLC25A1 shRNAs. **c** OCR rates of the indicated tumor cells in vehicle (black bars) or CTPI-2 treated (gray bars) cells. In all cases, treatment with CTPI-2 was for 3 h and OCR levels were normalized to cell number. There were no changes in viability during this period of time. Bars represent the standard deviation, asterisks refer to **P ≤ 0.01; ***P ≤ 0.001; ****P ≤ 0.0001 by unpaired T-test

We have previously proposed that SLC25A1 promotes mitochondrial respiration [27]. To enlighten the mechanisms by which SLC25A1 affects CSC metabolism, we measured oxygen consumption rates (OCRs) of monolayer or sphere cultures with or without SLC25A1 overexpression with the Seahorse analyzer. In this system, the sequential injection of inhibitors of the electron transport chain (ETC) allows measurement of basal OCR (BR), as well as of maximal respiration (MR) [31]. The difference between maximal and basal OCR is defined as spare respiratory capacity, SRC. Although normal cells operate at a basal level of OCR, the SRC provides ATP under stress conditions that enhance the cellular energetic demand and its exhaustion has been linked to induction of apoptosis, to neurodegeneration and to heart disease [32, 33]. Relatively to two-dimensional (2D) cultures, H1299 spheres exhibited higher OCR levels (Supplementary Figure 4a) and SLC25A1 overexpression enhanced basal, SRC and mitochondrial ATP output (Fig. 4a). Noticeably, SLC25A1-expressing spheres displayed higher maximal mitochondrial respiration following incubation with the mitochondrial uncoupler Carbonyl cyanide-4-(trifluoromethoxy)phenylhydrazone (FCCP), indicative of enhanced electron flux across the ETC. As expected, in cells expressing the SLC25A1-shRNAs, OCR levels were severely suppressed (Fig. 4b).

**Fig. 5:**
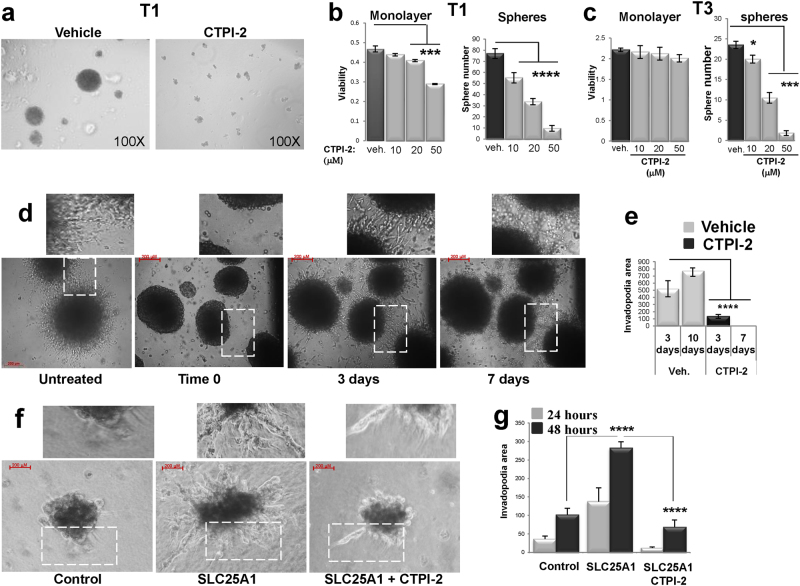
SLC25A1 promotes matrix invasion. **a**–**c** Representative results showing that CTPI-2 disrupts self-renewal and sphere-forming capacity of the indicated tumor cells relative to monolayer cultures. **d**, **e** Spheres embedded in a collagen-based matrix were imaged immediately after plating (time 0) and for 7–10 consecutive days. Fields were identified by numbers on the bottom of the plate. The white rectangles indicate the enhanced areas of each field shown at the top. Invadopodia formation was calculated by first measuring the areas of the spheres (inner area) and the area of the spheres along with its invadopodia (outer area). The percent difference between the outer area and the inner area was graphed and spheres of similar areas were selected for comparison, so to exclude the influence of different cell number. **f**, **g** Similar experiments were performed in SLC25A1-expressing cells.Bars represent the standard deviation, asterisks refer to *P ≤ 0.05; ***P ≤ 0.001; ****P ≤ 0.0001 by unpaired T-test

We next expanded these experiments to study the effects of CTPI-2 in patient-derived NSCLC tumors, named T1, T2 and T3. Consistent with previous results, spheres derived from these tumors were enriched in stem cell markers (nearly 100%) and displayed higher OCR levels relative to monolayer cultures (Supplementary Figure 4bc). CTPI-2 inhibited mitochondrial respiration of CSCs spheres (Fig. 4c) and effectively inhibited their self-renewal again showing lesser anti-proliferative activity in 2D cultures (Figs. 5a–c).

CSCs become migratory and acquire the ability to invade the extracellular matrix as a critical step for metastatic dissemination [34, 35]. We found that SLC25A1 expression is elevated in metastatic lymph nodes (Fig. 1), suggesting a role for this protein in invasion. We tested this idea by employing matrix invasion assays [36]. Spheroid CSC cultures were first grown in low attachment plates and in the absence of serum for three passages and were next embedded in the collagen matrix, followed by treatment with CTPI-2. The results showed that T1 spheres were highly invasive and CTPI-2 completely reverted this phenotype (Fig. 5d). Similarly, SLC25A1 expression in H1299 cells enhanced the invasive ability of CSC spheres, and CTPI-2 again severely inhibited this phenotype (Figs. 5f, g). Collectively, these results show that CTPI-2 has broad activity on lung CSCs, inhibiting mitochondrial respiratory activity, self-renewal and matrix invasion.

### An SLC25A1-mediated citrate import pathway supports mitochondrial respiration and oxidative balance in CSCs

Citrate plays essential activities in modulating the cellular energetic state. The sub-cellular pool of citrate is not static and albeit the best-known activity of SLC25A1 consists of promoting the export of citrate from the mitochondria to the cytoplasm, a reverse citrate import pathway has also been documented during matrix detachment [37, 38]. In the cytoplasm, citrate is the precursor for lipid synthesis, whereas in the mitochondria it is utilized for the Krebs cycle via oxidative phosphorylation.

**Fig. 6:**
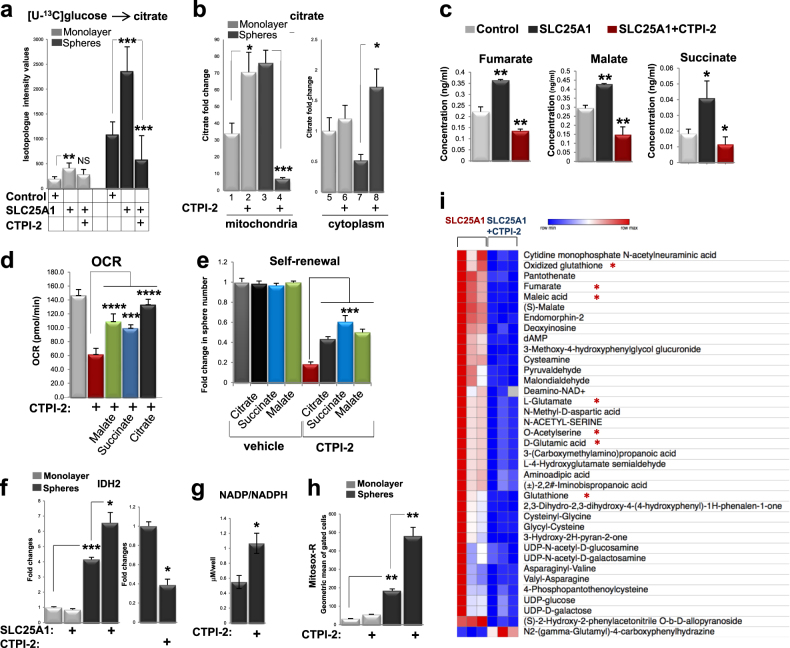
SLC25A1 is a key regulator of the citrate pool and redox balance. **a** Mass spectrometry analysis of citrate in monolayer of sphere cells labeled with [U-^13^C]glucose. **b** Assessment of citrate levels in mitochondrial or cytoplasmic fractions and in the presence or absence of CTPI-2, added to the media for four hours before the assay. **c** Levels of the indicated metabolites assessed with LC-MS in the indicated cell lines grown as spheres. **d** OCR was assessed in untreated or CTPI-2-treated spheres in the presence or absence of membrane permeable methyl-malate, succinate, or citrate over the course of a 3-h period. **e** Self-renewal in H1299 cells grown as single cells in semisolid media after 1 week of treatment with the indicated metabolites in mock treated or CTPI-2 treated cells. All metabolites were re-added to the culture media every 48 h. **f**–**h** qRT-PCR of the IDH1 mRNA **f**, assessment of NADP/NADPH ratio **g** and of superoxide levels detected with mitosox red **h**, in the indicated conditions. **i** A *Heatmap* comparison of the changes in metabolite levels between cells expressing SLC25A1 untreated or treated with CTPI-2 is shown. Asterisks indicate metabolites most relevant to the pathways analyzed in this study

To determine whether changes in SLC25A1 export/import activity account for the induction of mitochondrial respiration in CSCs, we first studied glucose-derived citrate levels in monolayer or spheres expressing SLC25A1 or treated with CTPI-2 with U^13^-C-glucose labeling experiments. This analysis showed that glucose-derived citrate was enriched in SLC25A1 spheres, suggesting that SLC25A1 directs the flux of glucose toward citrate synthesis, an activity that was reversed by CTPI-2 (Fig. 6a). Furthermore, sub-fractionation experiments showed that a short pulse treatment with CTPI-2 induced accumulation of citrate in the mitochondria in monolayer cultures (Fig. 6b, lanes 1 and 2), but decreased the mitochondrial citrate pool in spheres, leading to concomitant accumulation in the cytosol (Fig. 6b, lane 4 vs. 3 and lane 7 vs. 8; see Supplementary Figure 5a for the fractionation experiments).

These data support the idea that while in differentiated 2D cultures SLC25A1 predominantly exports citrate out into the cytosol, as indicated by the increase of the mitochondrial pool induced by CTPI-2, in CSCs spheres the reverse import from the cytoplasm to the mitochondria is favored, hence the drop of the mitochondrial pool of citrate induced by CTPI-2 and its increase in the cytosol. This import activity can explain the SLC25A1-induced mitochondrial respiration and might account for the effects of CTPI-2. In the mitochondria, citrate is converted into essential metabolites for intermediary carbon metabolism, including malate and succinate, which enter the Tricarboxylic Acid Cycle (TCA) cycle. Therefore, a deficit in the SLC25A1 import function could reduce the efficiency of the cycle. Consistent with this speculation, SLC25A1 increased the levels of fumarate, malate and succinate, and CTPI-2 starkly reverted this effect (Fig. 6c). Moreover, the addition of citrate, succinate or malate rescued mitochondrial respiratory capacity and partially restored self-renewal in CTPI-2-treated spheres (Figs. 6d, e). Thus, SLC25A1 promotes oxidative metabolism in the mitochondria and this activity is especially favored in CSCs.

Mitochondrial oxidative metabolism needs the balance of anti-oxidant systems to neutralize reactive oxygen species (ROS) [37, 39, 40]. The Tricarboxylic Acid Cycle (TCA) cycle generates NADPH via the action of mitochondrial isocitrate dehydrogenase-2 (IDH2) that catalyzes the oxidative decarboxylation of isocitrate, converting Nicotinamide adenine dinucleotide phosphate (NADP^+^) to Nicotinamide adenine dinucleotide phosphate (NADPH). NADPH reduces glutathione, providing a buffering system for mitochondrial ROS. As shown in Fig. 6f, IDH2 mRNA levels were enhanced by SLC25A1 and reduced by CTPI-2. Additionally, CTPI-2 increased the NADP^+^/NADPH ratio and induced a strong build-up of mitochondrial superoxide (Figs. 6g, h).

**Fig. 7:**
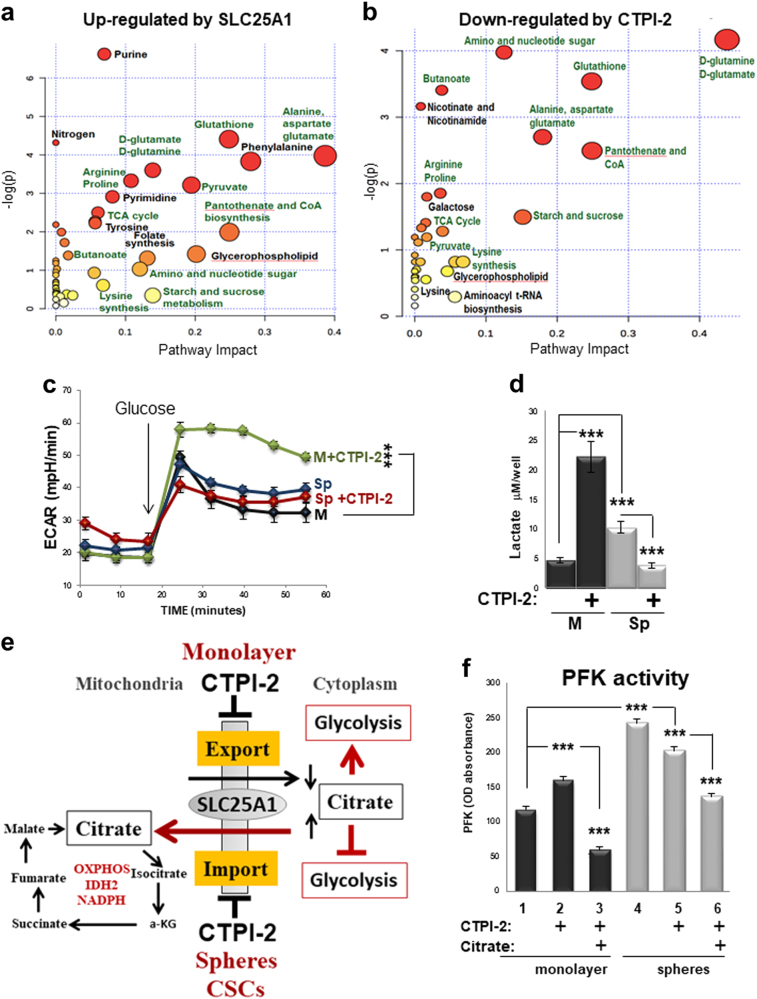
Metabolic pathways affected by SLC25A1. **a**,** b** Metabolomic analysis with LC-MS of SLC25A1-expressing spheres versus control spheres **a** and of SLC25A1-expressing spheres untreated versus treated with CTPI-2 **b**. In **a**, 1412 metabolites were quantified, 181 of which showed statistically significant changes (*p* < 0.05). In **b**, 413 metabolites were quantified and 91 metabolites showed statistically significant changes (*p* < 0.05). Analysis was performed with Morpheus, Metaboanalyst and Ingenuity Pathway analysis (IPA). One minus Pearson correlation’ was used for hierarchical clustering of the metabolites. Panel** a** shows metabolites induced by SLC25A1 relative to control cells, and panel **b** shows metabolites that were downregulated by CTPI-2 in SLC25A1-expressing spheres. Pathways that are common in the two comparisons are highlighted in green. **c**, **d** Extracellular acidification rates (ECAR) and lactate levels in the indicated growth conditions and in the presence or absence of CTPI-2. **e** Proposed model for the effects of SLC25A1 and CTPI-2 in monolayer and spheres (see also text for explanation). **f** Phosphofructokinase (PFK) activity was assessed in cells first grown in monolayer or spheres for 36 h and then treated with citrate (10 mM) overnight. The following day, 5 mM citrate was re-added to the media and cells were either mock treated (lanes 1, 4) or treated with CTPI-2 (lanes 2, 3, 5, 6) for 3 h, before quenching and assessment of PFK activity. Bars represent the standard deviation, asterisks refer to ***P ≤ 0.001 by unpaired T-test

To obtain a further unbiased view of the activities exerted by SLC25A1 and CTPI-2 on CSC metabolism, we next performed metabolomic analysis of SLC25A1-expressing spheres with or without CTPI-2 treatment by using Liquid Chromatography - Mass Spectrometry (LC-MS). First, the most represented metabolites in SLC25A1 spheres were oxidized glutathione and components of the TCA cycle (fumarate, maleic acid, S-malate), and these metabolites were reduced by CTPI-2 (Fig. 6i). Noticeably, amino acids derived from the TCA cycle [41, 42], particularly aspartate, alanine and glutamine, were also upregulated by SLC25A1 (Figs. 7a, b). Moreover, nearly all of the metabolic pathways upregulated in SLC25A1 spheres were downregulated by CTPI-2, further demonstrating the specificity of this drug (Figs. 7a, b). We conclude that SLC25A1 is a key regulator of the sub-cellular compartmentalization of the citrate pools, enabling CSCs to utilize citrate for mitochondrial respiration and leading to the activation of IDH2-NADPH system to alleviate the harmful effects of ROS build-up.

### CTPI-2 is a unique regulator of glycolysis that limits the metabolic plasticity of CSCs

Cancer cells switch toward aerobic glycolysis to derive energy when mitochondrial activity is inhibited [19, 43]. The anti-diabetic drug metformin, a complex I inhibitor, displays antitumor activity against pancreatic CSCs but resistance to metformin occurs, in part, through activation of glycolysis [19]. Therefore, we studied how CTPI-2 affects glycolytic rates. First, we found that extracellular acidification rates (ECAR) and lactate levels, which are indicative of glycolysis, were induced in 2D cultures treated with CTPI-2 (Figs. 7c, d). In agreement with the switch toward oxidative mitochondrial metabolism, glucose was diverted toward mitochondrial respiration in spheres (Supplementary Figure 5b) and lactate levels were elevated in spheres (Fig. 7d), likely consistent with its recently discovered role as a carbon source for the TCA cycle [44]. By contrast, ECAR and lactic acid levels were slightly diminished by CTPI-2 in spheroid conditions (Figs. 7c, d), an effect opposite to that of metformin that, as expected, enhanced glycolytic rates (Supplemental Fig. 5b). Thus, CTPI-2 regulates glycolysis in an opposite fashion in differentiated monolayer cultures versus CSC spheres.

Because cytoplasmic citrate is a negative allosteric regulator of the glycolytic enzyme Phosphofructokinase (PFK) [45], we hypothesized that this unique mode of action relies upon CTPI-2 ability to differentially regulate the cytoplasmic concentration of citrate, which is lowered in monolayer cultures but increased in spheres, thus allowing citrate to maintain a sustained block on glycolysis in the latter but not in the former (depicted in Fig. 7e). Supporting this hypothesis, CTPI-2 enhanced PFK activity in monolayer cultures and such enhancement was entirely reversed by citrate (Fig. 7f, lanes 3 vs. 2). In contrast, PFK activity was reduced in spheres (lanes 4 and 5).

Based on these data, we propose that CTPI-2 inhibits mitochondrial respiratory activity without inducing a compensatory increase in glycolysis in CSCs. Therefore, while with other mitochondrial inhibitors CSCs may escape the block of oxidative phosphorylation by deriving energy through this pathway, such switch is prevented by CTPI-2 and hence, this drug is more effective in limiting the energetic supplies of this cell population.

### CTPI-2 is synthetic lethal with cisplatin or the EGFR inhibitor AZD9291

Drug resistance has been attributed the CSC population [9–13]. We therefore asked whether resistant tumors evolve toward a stemness-associated SLC25A1-dependent metabolic profile. To this end, we employed the patient-derived NSCLC tumor samples. The T1 tumor does not harbor “targetable” mutations and was derived from a patient who progressed under platinum doublet chemotherapy. The T2 tumor carries the L858R/T790M mutation and the patient progressed on dacomitinib, a second-generation EGFR inhibitor. The T4 tumor also harbors the L858R/T790M and the patient progressed on osimertinib (or AZD929), a third-generation EGFR inhibitor that targets the T790M mutation [46–49].

**Fig. 8:**
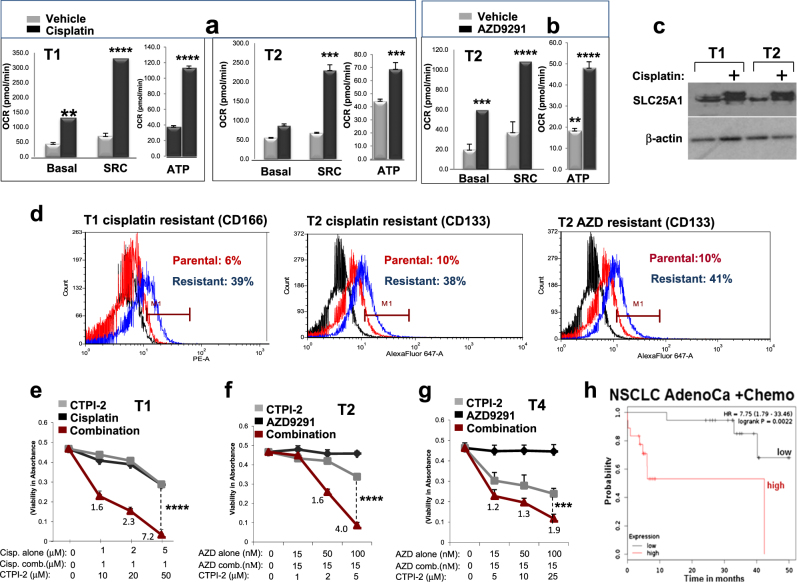
SLC25A1-dependent mitochondrial respiration drives therapy resistance. **a**,** b** T1 and T2 cells were treated with cisplatin (0.5–1 μM) or AZD9291 (1 μM), as indicated in each panel, passaged in the presence of the drugs several times, then subjected to OCR analysis with the Seahorse analyzer. **c** SLC25A1 protein levels in T1 and T2 cells cultured in the presence or absence of cisplatin. **d** FACS analysis of CD166 and CD133 markers in the cisplatin or AZD9291-resistant T1 or T2 cells. **e**–**g** T1 **e**, T2 **f** and T4 **g** cells were untreated or treated with the indicated drugs. The concentration of drugs employed is indicated at the bottom of each panel. Viability was assessed with Crystal Violet after 5 days of treatment. Relative (*R*) index calculations were used to assess the type of drug interactions and are indicated in each panel. The R index is calculated as the expected cell survival (*S*_exp_; the product of relative survival in cisplatin and relative survival in CTPI-2) divided by the observed relative survival in the presence of both drugs (*S*_obs_). *S*_exp_/*S*_obs_ = 1.0 denotes an additive interaction, whereas >1.0 denotes a synergistic interaction, *R* index values approaching 2.0 are indicative of strong synergy. **h** Kaplan–Meier survival curves relative to SLC25A1 expression in lung AdenoCa patients that received chemotherapy. Red and black lines indicate high and low SLC25A1 expression, respectively. Analysis was performed by using the Km plotter database (http://kmplot.com/analysis/). Bars represent the standard deviation, asterisks refer to **P ≤ 0.01; ***P ≤ 0.001; ****P ≤ 0.0001 by unpaired T-test

For these experiments, we used cells grown as monolayer because they better represent the “bulk” heterogeneous population of cells exposed to drugs [50]. T1 or T2 cells were treated chronically with cisplatin or AZD9291 until they could tolerate twice the IC50 values. Treated cells slowed proliferation rates (data not shown) and underwent a dramatic shift toward mitochondrial respiration, which improved mitochondrial-derived ATP production, paradoxically indicating a better energetic output under drug treatment (Figs. 8a, b). In the case of cisplatin, SLC25A1 expression levels were increased (Fig. 8c). Further, all resistant cells were enriched for stem cell markers (Fig. 8d), arguing that the stem cell phenotype is coupled to mitochondrial respiratory activity. Most importantly, CTPI-2 re-sensitized the T1, T2 and T4 cells to cisplatin or AZD9291 treatment in a synergistic fashion (Figs. 8e–g). Mechanistically, although treatment with cisplatin or CTPI-2 alone did not induce apoptosis, a strong pro-apoptotic effect occurred when cells were co-treated with both drugs (Supplementary Figure 6a). Given that H1299 cells harboring the SLC25A1 knock-down were hyper-sensitive to cisplatin (Supplementary Figure 6b), these results strongly suggest a synthetic lethal activity of CTPI-2 with cisplatin treatment. Furthermore, SLC25A1 levels negatively affect survival in NSCLC patients treated with chemotherapy (Fig. 8h), hence strengthening the relevance of our findings.

In light of these data, we propose that under drug treatment certain subtypes of lung cancer cells switch toward a dormant, stemness phenotype in which they depend upon SLC25A1-mediated mitochondrial respiration, and this metabolic trait allows them to endure the stress of drug treatment in an energetically favorable state. Consequently, in such state they become most vulnerable to SLC25A1 inhibitors.

### CTPI-2 inhibits tumor growth in in vivo models of NSCLC

**Fig. 9:**
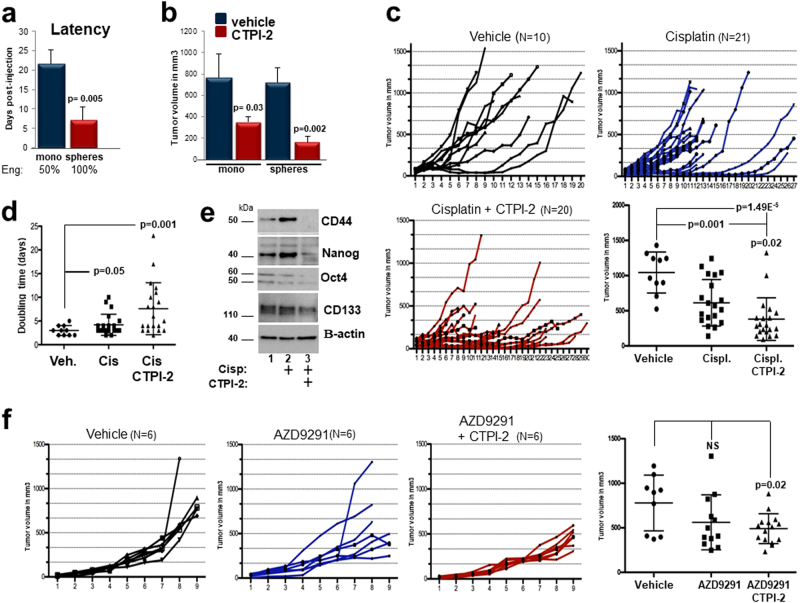
Antitumor activity of CTPI-2. **a** T1 cells were grown as monolayer or spheres were injected in Balb/c athymic nude mice at 5 million cells/injection site. The time at which tumors started to appear was recorded and plotted in the graph (*n* = 7 per group). Once tumors were fully established there were no differences in tumor volumes between the two groups (see **b**). **b** Tumor volumes of monolayer (blue bars) or spheres (red bars) treated with vehicle (DMSO) or CTPI-2 (*N* = 6 per group). CTPI-2 was administered at 26 mg/kg at alternate days intra-peritoneally. **c** T1 cells were injected in immunocompromised mice, which were randomized to receive vehicle, cisplatin (3 mg/kg every 3 days), alone or in combination with CTPI-2. The figure shows the growth curves of all tumors in each treatment group. The number of tumors is indicated at the top of each panel. The graph shows a dot plot analysis of all tumors volumes at the end of the experiments. Each point represents the mean ± SD of tumor volumes. Statistical significance was assessed using both unpaired, two-tailed Student's *t-*test and analysis of variance (ANOVA). **d** Tumor doubling times were calculated by using the formula employed by radiologists. An online calculator was used at: http://www.chestx-ray.com/index.php/calculators/doubling-time, by analyzing the difference between tumor volumes the day before starting each treatment and at the end of the experiment. **e** Cell extracts derived from T1 tumors treated with vehicle (1) or cisplatin (lane 2) or combination (lane 3), were processed in immunoblot with the indicated antibodies. The position of each band relative to the molecular weight marker is indicated. **f** T2 cells were injected in immunocompromised mice. When tumors reached a size of 30–50 mm^3^ mice were randomly assigned to either control vehicle, or AZD9291 treatment (10 mg/kg every other day) or combination treatment with CTPI-2 (*n* = 6 tumors per group). Individual growth curves for all tumors in each treatment group are shown and the end-tumors are analyzed in a dot plot

We next studied the therapeutic potential of CTPI-2 by injecting T1 cells either as monolayer or spheres into nude mice. Monolayer T1 cultures resulted in engraftment rates of 50% and tumors arose within 3 weeks, compared with spheres that instead generated tumors with 100% frequency, and at much shorter latency (Fig. 9a), consistent with the enrichment of CSCs with tumor-initiating capability herein. In agreement with our previous data, CTPI-2 more effectively inhibited the growth of sphere-derived T1 tumors relative to monolayer cultures (Fig. 9b). Importantly, CTPI-2 was well tolerated during the entire treatment period.

We next studied CTPI-2 in combination therapy with either cisplatin or AZD9291 (Fig. 9c). In the cisplatin-treated group, many T1 tumors were either overtly resistant to the treatment or exhibited growth rates similar to control, indicating that tumor growth was delayed but not completely inhibited. In contrast, in the co-treatment group there were only two resistant tumors, but many tumors showed clear regression remaining stable for a longer time. Accordingly, CTPI-2 significantly extended the tumor doubling times compared with cisplatin (Fig. 9d). Importantly, cisplatin enriched for stemness markers and CTPI-2 completely reverted this effect, in agreement with our previous findings (Fig. 9e). Consistent with the in vitro data (Fig. 8), co-treatment with CTPI-2 and AZD9291 also led to a prominent tumor growth inhibition relative to AZD9291 treatment alone (Fig. 9f).

## Discussion

The major reason of mortality in lung cancer is the emergence of resistance. Resistance to platinum-derived agents represents a significant clinical challenge because these drugs are very often used as first-line therapy in tumors that do not harbor targetable mutations, or as rescue therapy in tumors that have become resistant to targeted agents [1, 2]. Similarly, resistance to first-, second- and third-generation EGFR inhibitors is a major cause of mortality in patients harboring EGFR mutations [46–49]. As the genetic spectrum of mutations continues to evolve during therapy, the development of treatments that act regardless of the tumor mutational profile is an attractive concept. Noticeably, immune-checkpoint modulators act relatively independently of the driver genetic alterations currently being the most efficacious therapeutics [3].

We have shown that SLC25A1 plays a key role in the adaptive mechanisms that allow some tumor cells to acquire drug resistance. This finding raises the attractive possibility that common metabolic features involving a switch toward SLC25A1-driven mitochondrial metabolism hallmarks resistance to different therapies. In fact, the slow-replicating, quiescent state of CSCs conceivably allows tumor cells to tolerate anti-proliferative signals conveyed by most therapeutics, protecting them from cell death, whereas the high energetic output derived from mitochondrial oxidative metabolism ensures survival. As CSCs eventually exit the quiescence/dormancy state reigniting recurrence, the idea that these cells could be eradicated therapeutically when they are still in a quiescent state is very attractive. Attacking the very foundation for their survival, the metabolism, is feasible given the rapidly expanding repertoire of metabolic inhibitors, to which we have now contributed with the discovery of CTPI-2. Thus, we propose, and will further test the idea, that SLC25A1 inhibitors render tumors with a broad range of mutational landscapes better manageable.

We found that SLC25A1 is overexpressed in most lung cancers relative to normal tissues and in metastatic sites. The finding that CTPI-2 blunts proliferation in non-CSCs, although to a significantly lesser extent relative to CSCs, may indicate that SLC25A1 is not a specific target for CSCs, which represent a discrete tumor sub-population. However, other pathways previously advocated for the development of CSC therapeutics, including Notch, Hedgehog and Wnt/b-catenin, are active also in highly proliferating cancer cells [51–53].

Our results establish that SLC25A1-induced mitochondrial metabolism promotes the development of the therapy resistant, stemness phenotype at least in some types of NSCLC. Nevertheless, other metabolic pathways most likely also contribute to this phenotype. Recent studies have demonstrated the importance of reductive carboxylation of glutamine, via IDH1, which implements the production of cytoplasmic citrate, in a model system of matrix detachment [37]. Although the experimental conditions in that study were significantly different from ours, in that they did not enrich the CSCs population, we have found that SLC25A1 enhances the mRNA expression levels of IDH1 (data not shown). Therefore, it is possible that SLC25A1 activity in CSCs also relies upon IDH1 activity. Other important metabolic pathways required for NSCLC also involve de novo lipid synthesis [54], which depends upon SLC25A1-mediated citrate export.

In summary, our studies have shown that SLC25A1 is an essential component of the tumor cell metabolism and have enlightened novel mechanisms and therapeutic perspectives for the treatment of resistant NSCLC tumors.

## Materials and methods

### Cells, reagents, antibodies, primers

The H1299 cell lines and other cell lines employed in this study were obtained from ATCC or from the tissue culture core facility at LCCC. Cells were grown in Dulbecco’s modified Eagle’s medium (DMEM, 25 mM glucose, with glutamine and pyruvate from Invitrogen) and supplemented with 10% fetal calf serum. The reagents used in this study were: 1,2,3, Benzenetricarboxylica acid (BTA), Sigma (# B4201), CTPI-1 was synthesized by Dr. Mikell Paige, but is also available from Sigma, CAS number 412940-35-3. The CTPI-2 is listed in the Pubchem (CAS#: 68003-38-3) and it was purchased from Enamine Ltd or from Santa Cruz. The SLC25A1-specific shRNA vectors were purchased from Sigma (TRCN0000232825; TRCN0000255350). The vector-expressing human SLC25A1 untagged or Flag-Myc epitope tagged were from Origene (#SC120727 and RC200657, respectively). The anti-SLC25A1 antibody used in immunoblot was from Santa Cruz Biotech, # sc-86392 employed at 1:1000 dilution. Citrate, lactate and PFK measurements were performed by using a commercially available kits (Biovision, Bioassays).

### Growth of monolayers and sphere cultures

Tumor cell lines were grown as monolayer in complete Dulbecco’s modified Eagle’s media (DMEM, Gibco; supplemented with 10% fetal bovine serum and 1% of 5000 units/ml of penicillin–streptomycin (pen–strep), Gibco). To generate spheroids, cells were grown in falcon bacteriological Petri dishes coated with 2% poly(2-hydroxyethyl methacrylate) dissolved in 100% ethanol. The growth medium was DMEM/F12, Gibco; supplemented with 20 ng/ml of epidermal growth factor (EGF), 5 ng/ml of fibroblast growth factor, 0.375% 100 × N2 supplements (Gibco) and 1% pen–strep. Monolayer cells were dissociated using 0.25% Trypsin-EDTA (Gibco), whereas spheroid cultures were dissociated using StemPro Accutase (Gibco). Patient-derived cell lines were grown as described in the primary lung cultures section.

### Self-renewal assays

Tumor cells cultured as spheroids were dissociated and diluted in 2 × sphere media containing 1% methylcellulose, which was previously dissolved in cold DMEM/F12 media. Either 200, 500 or 1000 cells were the plated in each 96 well and were incubated in the presence or absence of drugs, as indicated. The spheres were allowed to form for a week–10 days at which point they were counted. Six biological replicates were used for quantification of the results.

LC-MS methods cell extracts from unlabeled cells or from cells labeled with 10 mM [U-^13^C]glucose were pelleted and re-suspended in 150 µl of water and lysed by heat shock treatment by plunging the tubes into dry ice for 30 s and immediately into 37 °C water batch for 90 s followed by sonication for 30 s. Subsequently, 600 µl of chilled 100% methanol containing internal standards (10 µl of 1 mg/ml debrisoquine and 50 µl of 1 mg/ml 4-nitrobenzoic acid added to 10 ml of 100% methanol) was added, followed by the addition of 600 µl of chloroform. The samples were centrifuged at 13,000 rpm for 20 min at 4 °C. This resulted in two phases, which were carefully separated, followed by the addition of 100% chilled acetonitrile and overnight incubation at −20 °C. The samples were centrifuged at 13,000 rpm for 20 min at 4 °C and supernatant was vacuum dried. The dried samples were re-suspended in 50% methanol in water for UPLC- QT of analysis. In all, 2 µl of each sample was injected onto a Waters Acquity BEH C18 1.7 µm, 2.1 × 50 mm column using an Acquity UPLC system by Waters Corporation, Milford, MA. The gradient mobile phase consisted of Solvent A—100% water with 0.1% formic acid, Solvent B—100% acetonitrile with 0.1% formic acid and Solvent D—90% Isopropanol and 10% acetonitrile with 0.1% formic acid. The column temperature was set to 40 °C and flow rate of 0.4 ml/min. The gradient started with 95% of Solvent A and 5% of solvent B at a ramp of curve 6. At 8 min, it shifted to 98% Solvent B and 2% Solvent D and stayed until 11 min. From 12 min, the gradient moved to its initial conditions of 95% of Solvent A and 5% of solvent B at a ramp of curve 6. The total run time was 13 min. The elution from the column was introduced to Quadrupole Time of flight Mass spectrometer (Waters G2- Qtof) by electrospray ionization in both positive and negative mode. The capillary voltage was 3.0 kV for positive mode and 1.50 kV for negative mode. The sampling cone voltage was at 30 V. The source temperature was set to 120 °C and desolvation temperature to 500 °C. The cone gas flow was maintained at 25 liter/h and desolvation gas flow at 1000 liter/h. Leucine–encephalin solution in 50% acetonitrile was used a reference mass ([M + H]^+^ = 556.2771 and [M-H]^−^ = 554.2615). The data were acquired in centroid mode from mass range of 50–1200 with the software Mass lynx (Waters Corporation). Pooled quality controls samples were injected after every 10 injections. The raw data files were imported in the MAVEN software environment for identification of metabolites that showed incorporation of 13-C label.

### Gene expression profiling of CTPI-2-treated or SLC25A1 shRNA-expressing cells

Total RNA was isolated from cell pellets using the Direct-zol RNA MiniPrep kit (Zymo Research, USA). The RNA quality and quantity was estimated by UV-VIS spectrophotometry using the NanoDrop ND-1000 spectrophotometer. RNA integrity was assessed using the Agilent RNA 6000 Nano Kit on the Agilent 2100 Bioanalyzer to calculate DV200 (the fraction of RNA molecules >200 nt). Final RNA yield and concentration was measured using the Qubit RNA HS Assay Kit, and subsequently normalized to the same concentration across all samples before input. Gene expression profile data were generated using one-color chemistry on the Agilent SurePrint G3 Human Gene Expression v3 Microarray Kit, 8 × 60 K platform according to the manufacturer’s standard protocol. Raw data (.tiff files) generated after scanning the array on the Agilent G2505C DNA Microarray Scanner was processed with Agilent Feature Extraction software v11.5.1.1, which provides QC Metric Sets with thresholds to interrogate normal ranges for key metrics. The Feature Extraction output “.txt” files, which is considered as raw data, were loaded into the Partek Genomics Suite for data analysis. Analysis of variance (ANOVA) model was utilized to do a global test across three groups: control PLKO, SLC25A1-shRNA, and Control plus CTPI-2. And three pair-wise comparisons were made. Fold changes, raw p-values, and Benjamini–Hochberg false discovery rate adjusted *p-*values were calculated. For the three pair-wise comparisons, the cutoff of “raw *p*-value < 0.05 and absolute value of fold change >2” was used to get three lists of significant genes correspondingly. Normalization and correction of array-wise background level was conducted in R using the *limma* package. Briefly, the normal-exponential convolution method was used with maximum likelihood estimation to correct background levels. An estimate of overlap probability was generated using a hypergeometric test.

### Primary lung cancer cultures

Surgical samples were minced and digested with dispase (50 U/ml) in DMEM media containing 5% serum for 1 h. Pleural effusions were centrifuged, washed once in phosphate-buffered saline (PBS) and then plated. All primary cultures were initially grown on six-well plates pre-coated with 2% Geltrex (Life Technologies) and 1% Maxgel (Sigma Aldrich). Unlike Geltrex, Maxgel is a humanized matrix, native and non-denatured. We established that the inclusion of Maxgel in the system enhances the initial attachment of primary tumor cells, likely because Maxgel povides a more natural ECM conformational substrate. The day after plating, the media containing unattached cells, was typically transferred to a fresh plate, in most of cases generating new attached cultures. The media (Stem Cell media) is the DMEM/F12 (Thermo-Fisher # 11320-033 basic media) supplemented with the following components: insulin-transferrin-sodium selenite (ITS; 1:1000; Sigma # I1884); glutamine (Thermo-fisher) to 4 mM final concentration; sodium pyruvate (Thermo-Fisher) to 2 mM final concentration; Rock Inhibitor (Y-27632) 10 µg/ml; 2–5% knock out serum replacement (KNOSR, Thermo-Fisher #10828-028); 0.5% ALBUMAX (lipid-enriched bovine serum albumin; Thermo-Fisher #11020-021); N2 supplement (Thermo-fisher) 1:250; EGF (5 ng/ml; Peprotech); human Fibroblast Growth Factor (hFGF) (20 ng/ml; Bechman); Insulin like Growth Factor - 1 (IGF-1) (Peprotech; 10–20 ng/ml). The matrix was replaced every 2 days.

### Cellular proliferation and cyto-toxicity assays

The proliferative capacity of cells was assessed by plating cells in triplicate at a concentration of 2000–10,000 cells/well in 96-well plates in triplicate with or without drug treatment for 48 h. The cells were then washed with 1 × PBS and fixed with cold 100% methanol in −20 °C for 20 min. Methanol was washed off and the cells were stained with 0.5% crystal violet dissolved in 25% methanol and water at 4 °C for 20 min. The staining was washed off using Deionized water (DI) water and the plate was dried overnight. On day 2, 100 mM sodium citrate in 50% ethanol and 50% water was added to the wells and absorbance at 550 nm was measured and corrected with readings at 405 nm. In other experiments, cell viability was assessed by Trypan blue exclusion and visual count of the live cells.

### Flow cytometry

Tumor cells were trypsinized and washed in 1 × PBS. The cells were then stained with the antibodies of choice: CD166-PE (Biolegend, 343904) or CD133–647 (Biolegend, 141216) or CD44 (Biolegend, 103047); in 1% BSA(Bovine Serum Albumin)-PBS. Cell cycle of monolayers or spheres of the cells were analyzed by fixing the cells in cold 75% ethanol. The analysis was carried out by the core facility of LCCC at Georgetown University. The annexin V (Biolegend, 640912) and propidium iodide (Biolegend, 421301) experiments were carried out following instruction from the manufacturer.

### Detection of citrate sub-compartmentalization

For the assessment of the citrate levels before collecting the cells, they were quenched using chilled 60% methanol and 0.85% ammonium sulfate solution for a few seconds. The cells were washed in PBS to wash out the methanol and then processed for mitochondrial isolation.

### Mitochondria isolation and cellular sub-fractionation

Mitochondria and cytoplasmic fractions were prepared as follows. Cells were collected in PBS for 5′, then the pellet was resuspended in five packed cell volumes of mitochondria buffer (250 mM sucrose; 20 mM HEPES (7.4); 10 mM KCl,1.5 mM MgCl_2_; 1 mM EDTA; 1 mM EGTA 0.5 M, supplemented with freshly added phenylmethylsulfonyl fluoride (PMSF) and 1 mM DDT). Cell lysates were passed through a 25G needle 10 times, then 5 times through a 27G needle, followed by incubation on ice for 10–15 min. Extracts were centrifuged at 720 *g* for 5 min. The supernatant was centrifuged again at 10,000 *g* and the derived pellet provided the mitochondrial fraction, the supernatant and the cytoplasmic fraction. To extract the mitochondria for measurement of citrate, the pellet from the previous centrifugation was re-suspended in PBS and subjected to three cycles of freeze-and-thaw in dry ice immersed in ethanol. The fraction was centrifuged again and re-suspended PBS. The supernatant (containing cytosolic and membrane fractions) was centrifuged multiple times to eliminate particulate material, and was used as the cytosolic fraction.

### Surface plasmon resonance

SPR experiments were performed on a Biacore 4000 instrument at room temperature at the Biacore Molecular Interaction Shared Resource of the Lombardi Comprehensive Cancer at Georgetown University. The NTA sensor chip surface was activated by injecting 0.5 mM NiCl_2_ for 1 min. Histidine-tagged recombinant purified SLC25A1 protein (0.07 mg/ml stock concentration) was used as the ligand to capture on the sensor surface. SLC25A1 was dissolved (1:50 dilution) in PBS-P (20 mM phosphate buffer (pH 7.4), 2.7 mM KCl, 137 mM NaCl and 0.05% surfactant P20) buffer and captured onto spots 2 and 4. SLC25A1 was captured to a level of ~3000. PBS-P was used as the capture running buffer. Small molecules were used as analytes to flow over the ligand immobilized surface. Spot 3 of all flow cells were used as reference. The calculated theoretical *R*_max_ was ~30 RU based on 1:1 interaction. The kinetics experiments were performed in the presence of HBS-P (10 mM HEPES (pH 7.4), 150 mM NaCl and 0.05% surfactant P20). The flow rate of all the solutions was maintained at 30 μL/min. The analyte concentrations were 0, 1.5216, 3.625, 6.25, 12.5, 25, 50 and 100 µM. Analyte concentrations showing signs of aggregation were excluded from the analysis. Data were analyzed by Biacore BiaEvaluation software for 1:1 steady-state binding model.

### Immunohistochemistry and analysis of tissue microarrays

The tissue microarrays were obtained from Biomax (LC10013at: lung adenocarcinoma with matched adjacent normal lung tissue; HLug-Ade060PG-01: human lung carcinoma with matched NAT; OD-CT-RsLug03–002: human lung carcinoma tissue with matched NAT). The SLC25A1 antibody for IHC was from Abcam (ab174924) used at 1:20 dilution. Visual scoring of the tissue-stained sections was done using a semiquantitative four-point scale, performed by one observer. This four-point scale is based on the degree of intensity of staining of the tumor cells and other tissues. These staining scores are represented in the pictures.

### Matrix invasion assays

Cells were first grown in low attachment plates and in the absence of serum for several days. When the first spheres formed, cultures were collected, spheres were allowed to precipitate by gravity in a 50 ml conical tube, after which time the spheres were gently pipetted several times to eliminate cellular aggregates. This was repeated three times and for three consecutive passages. Spheres were then embedded in a matrix composed of matrigel and collagen in wells that had been pre-labeled on the bottom to identify areas of interest. The formation of invadopodias was monitored on the same spheres at time 0 (immediately after plating) and for the following 7 days at 24-h intervals. The analysis of invasion was performed by using “imageJ”. Briefly, the area of invasion was measured by sketching an outline around the spheres using the freehand draw tool. The “measure” tool in “Analyze” on the top menu calculated the area for the selected spheroid. The areas of just the spheres (inner area) and the area of the spheres along with its invadopodia (outer area) were measured. The percent difference between the outer area and the inner area was graphed using excel to understand the extent of invasion in each cell type at different time points. Spheres of similar areas were selected, whereas the outliers were eliminated so as to compare the spheres of similar volumes.

### Statistical methods for drug interactions

Relative (*R*) index calculations were used to test the nature of the interaction between cisplatin, AZD9291 and CTPI-2. Briefly, the *R* index is calculated as the expected cell survival (*S*_exp_; the product of relative survival in cisplatin and relative survival in CTPI-2) divided by the observed relative survival in the presence of both drugs (*S*_obs_). *S*_exp_/*S*_obs_ = 1.0 denotes an additive interaction, whereas >1.0 denotes a synergistic interaction, *R*index values approaching 2.0 are indicative of strong synergy [45].

### Seahorse extracellular flux analyzer experiments

Cells derived from spheres or monolayer cultures were dissociated and plated overnight at 12,000 cells/well in DMEM with 10% FBS, 5 mM glucose, 1 mM pyruvate and 2 mM glutamine. For CTPI-2 or metformin treatments, the following day the medium was replaced with drug/vehicle, and treated for 3 h. The protocol was then followed as per the manufacturer’s instructions, briefly: for the mitochondrial stress test, the medium was replaced with DMEM without FBS or bicarbonate, containing 5 mM glucose, 1 mM pyruvate, 2 mM glutamine and placed in a CO_2_ free incubator at 37 °C for 1 h, transferred to the Xf96 extracellular flux analyzer (Agilent). The program consisted of three measurements of OCR/ECAR before the injection of each drug: oligomycin (0.5 μM final concentration), FCCP (2 μM) and rotenone/antimycin (0.5 μM of each). For the glycolysis stress test, cells were plated overnight as above, and the medium was washed twice the following day with DMEM without FBS, bicarbonate, glucose, glutamine or pyruvate, and incubated in this medium in a CO_2_ free incubator with/without drug treatments at 37 °C for 4 h. There were three measurements of OCR/ECAR before each injection: glucose (10 mM final concentration), oligomycin (0.5 μM) or 2-deoxyglucose (50 mM). For the analysis of glutamine oxidation, the protocol was the same as the glucose stress test, but with glutamine being the only compound injected (final concentration 4 mM) and OCR/ECAR measured for 60 min. When spheres were analyzed, they were dissociated with StemPro® Accutase® (Thermo Scientific) and plated under the same conditions as above as for the monolayers the day before the assay (H1299 spheres), or in stem cell media (See Stem Cell Media) with the wells coated in 15 μl of 2% geltrex diluted in DMEM:F12 (Thermo Scientific). The results were normalized to cell number *n*.

### Real-time PCR

Total RNA was isolated using Trizol® (Thermo Scientific), and 5 µg of total RNA was treated with DNase I (Thermo Scientific), in the presence of SUPERase^.^In^TM^ (Thermo Scientific) for 30 min at 37 °C; 5 mM EDTA was then added and the DNase I heat inactivated at 75 °C for 10 min. After adding 5 mM MgCl_2_, complementary DNA was generated using Superscript IV (Thermo Scientific) and random hexamers following the manufacturer’s instructions. Real-time PCR was carried out using PowerUp™ SYBR® Green Master Mix (Thermo Scientific), using the reference genes glyceraldehyde-3-phosphate dehydrogenase (GAPDH) and small nuclear ribonucleoprotein D3 (SNRPD3). The gene expression was normalized to the two reference genes and the relative gene expression fold changes calculated using the ∆∆CT method. Dissociation curves were analyzed and showed single amplification products to confirm the specificity of each primer pair, and RT samples were run to verify no genomic DNA contamination was present. The primer sequences were as follows:

GAPDH: forward: 5ʹ-CCCTCCGGGAAACTGTGGCG-3ʹ; reverse: 5ʹ-GCAGTGGGGACACGGAAGGC-3ʹ;

SNRPD3: forward: 5ʹ-GAGGACAACATGAACTGCCA-3ʹ; reverse: 5ʹ-TAACATGGGTGCGTTCTTCA-3ʹ;

Notch-1: forward: 5ʹ-GGTGAGACCTGCCTGAATG-3ʹ; reverse: 5ʹ-GTTCTTGCAGGGGGTGC-3ʹ;

KLF4: forward: 5ʹ-CACCATGGACCCGGGCGTGGCTGCCAGAAA-3ʹ; reverse: 5ʹ-AAGCTGACTTGCTGGGAACTTGACC-3ʹ; Oct2: forward: 5ʹ-AGATCAAGGCTGAAGACCCC-3ʹ; reverse: 5ʹ-GAGGAGCTGCTGTATGTCCC-3ʹ;

SLC25A1: forward: 5ʹ-CCCCATGGAGACCATCAAG-3ʹ; reverse: 5ʹ-CCTGGTACGTCCCCTTCAG-3ʹ;

Nanog (Bio-Rad, Cat # 10025636); Sox2 (Bio-Rad, Cat # 10025636).

### Modeling of *human* SLC25A1 and identification of SLC25A1 inhibitor

The *in silico* homology model for *human* SLC25A1 was derived from the Protein Model Portal of the PSI-Nature Structural Biology Knowledgebase (Uniprot ID: P53007). The docking pose for citrate, CTPI-1 and CTPI-2 were using UCSF Dock6.7 software. Briefly, following the rationale of Kaplan and co-workers, receptor spheres were generated using the program SPHGEN cover the area identified by Kaplan as Site 2 [43]. The ligand flexibility option was used for the docking and the top scoring conformation was minimized to give the final pose. Melting points of the identified compounds were determined in open capillary tubes on a Electrothermal melting point apparatus MEL-TEMP and are uncorrected. All NMR spectra were recorded on Bruker DRX spectrometer (400 MHz) spectrometer. NMR data were collected at 400 MHz for ^1^H and 100 MHz for ^13^C NMR. The chemical shifts are expressed in parts per million downfield from tetramethysilane as the internal standard in deuterated solvent and coupling constants (*J*) are in hertz (Hz). Data are reported as follows: chemical shift, integration, multiplicity (s is singlet, d is doublet, t is triplet, dd is doublet of doublets, m is multiplet) and coupling constants. All solvents and reagents were obtained from commercial suppliers and used without further purification. All evaporations were carried out in vacuo with a Büchi rotary evaporator. Thin-layer chromatography was performed on Merck silica gel plates 60F254 visualized by UV light at 254 nm or 310 nm. Flash chromatography was carried out using flash-grade silica gel 60 (40–63 µm, 230–400 mesh).

### Mice and treatments

To produce tumor xenografts, 5 × 10^6^ cells derived from either monolayer or sphere cultures were dissociated with Accutase, re-suspended in PBS and injected subcutaneously in the flanks of female balb athymic mice. Once detectable tumors started to form, their size was measured with a caliper in three dimensions and mice were randomized in the different treatment groups. Randomization was not blinded, but great attention was placed in ensuring its impartiality. For co-treatment experiments, mice were randomized to receive either PBS, or CTPI-2 at a concentration of 28 mg/kg, which was administered via intraperitoneal route at alternate days. Cisplatin was administered at 28 mg/kg every 3 or 4 days. AZD9291 was administered at 25 mg/kg every 3 days by oral gavage. Serial measurements were made every day after the identification of the initial tumor mass to determine growth curves in vivo. Tumor volumes were calculated using the formula for a prolate spheroid: volume = (4/3) × *a*^2^*b*, where *a* is the width and *b* is the length. All animals were sacrificed when the tumors exceeded 1.5 cm. At the completion of experiments, tumors were excised, weighed and statistical significance of differences in tumor volume were assessed. Animals were monitored once a week for the presence of signs of disease, particularly neurological disturbances or weight loss, and they were weighted periodically. All animal studies were approved by the Georgetown University Institutional Animal Care and Use Committee.

### Statistics

For the animal studies, power calculations for each treatment group were first determined by the Department of Biostatistics, Bioinformatics & Biomathematics at Georgetown University. The number of animals was calculated to detect a 50% difference at alpha = 0.05 and power = 0.8. Statistical significance was assessed using both unpaired, two-tailed Student's *t*-test andd ANOVA. Significant differences are indicated using the standard Michelin Guide scale (*P* < 0.05, significant; *P* < 0.01, highly significant; *P* < 0.001, extremely significant).

### Study approval of human subjects

All lung cancer specimens were obtained through IRB-approved protocols from the Center for Cancer Research (CCR)/NCI/NIH) and Georgetown University. All patients provided informed consent and their identity was blinded to the participants of this study.

## Electronic supplementary material

Supplementary Figures and Legends

## References

[1] Thomas A, Liu SV, Subramaniam DS, Giaccone G (2015). Refining the treatment of NSCLC according to histological and molecular subtypes. Nat Rev Clin Oncol.

[2] Subramaniam DS, Liu SV, Giaccone G (2016). Novel approaches in cancer immunotherapy. Discov Med.

[3] Gower A, Wang Y, Giaccone G (2014). Oncogenic drivers, targeted therapies, and acquired resistance in non-small-cell lung cancer. J Mol Med (Berl).

[4] Niederst MJ, Engelman JA, Hata AN. Distinct evolutionary paths to TKI resistance in NSCLC. Cell Cycle. 2016:1–2. 10.1080/15384101.2016.1221024 10.1080/15384101.2016.1221024 PMC5914907 27552405

[5] Thomas A, Giaccone G (2015). Why has active immunotherapy not worked in lung cancer?. Ann Oncol.

[6] Zakaria N, Satar NA, Abu Halim NH, Ngalim SH, Yusoff NM, Lin J, Yahaya BH (2017). Targeting lung cancer stem cells: research and clinical impacts. Front Oncol.

[7] MacDonagh L, Gray SG, Breen E, Cuffe S, Finn SP, O’Byrne KJ, Barr MP (2016). Lung cancer stem cells: the root of resistance. Cancer Lett.

[8] Eramo A, Lotti F, Sette G, Pilozzi E, Biffoni M, Di Virgilio A, Conticello C, Ruco L, Peschle C, De Maria R (2008). Identification and expansion of the tumorigenic lung cancer stem cell population. Cell Death Differ.

[9] Ku SY, Rosario S, Wang Y, Mu P, Seshadri M, Goodrich ZW, Goodrich MM, Labbe DP, Gomez EC, Wang J (2017). Rb1 and Trp53 cooperate to suppress prostate cancer lineage plasticity, metastasis, and antiandrogen resistance. Science.

[10] Wadosky KM, Ellis L, Goodrich DW (2017). Evasion of targeted cancer therapy through stem-cell-like reprogramming. Mol Cell Oncol.

[11] Hata AN, Niederst MJ, Archibald HL, Gomez-Caraballo M, Siddiqui FM, Mulvey HE, Maruvka YE, Ji F, Bhang HE, Krishnamurthy Radhakrishna V (2016). Tumor cells can follow distinct evolutionary paths to become resistant to epidermal growth factor receptor inhibition. Nat Med.

[12] Esendagli D, Gunel-Ozcan A (2017). From stem cell biology to the treatment of lung diseases. Curr Stem Cell Res Ther.

[13] Bertolini G, Roz L, Perego P, Tortoreto M, Fontanella E, Gatti L, Pratesi G, Fabbri A, Andriani F, Tinelli S (2009). Highly tumorigenic lung cancer CD133 + cells display stem-like features and are spared by cisplatin treatment. Proc Natl Acad Sci USA.

[14] Doherty MR, Smigiel JM, Junk DJ, Jackson MW. Cancer stem cell plasticity drives therapeutic resistance. Cancers. 2016;8. 10.3390/cancers8010008 10.3390/cancers8010008 PMC4728455 26742077

[15] Vander Heiden MG (2013). Exploiting tumor metabolism: challenges for clinical translation. J Clin Investig.

[16] Wang PY, Li J, Walcott FL, Kang JG, Starost MF, Talagala SL, Zhuang J, Park JH, Huffstutler RD, Bryla CM (2017). Inhibiting mitochondrial respiration prevents cancer in a mouse model of Li-Fraumeni syndrome. J Clin Investig.

[17] Tan AS, Baty JW, Dong LF, Bezawork-Geleta A, Endaya B, Goodwin J, Bajzikova M, Kovarova J, Peterka M, Yan B (2015). Mitochondrial genome acquisition restores respiratory function and tumorigenic potential of cancer cells without mitochondrial DNA. Cell Metab.

[18] LeBleu VS, O’Connell JT, Gonzalez Herrera KN, Wikman H, Pantel K, Haigis MC, de Carvalho FM, Damascena A, Domingos Chinen LT, Rocha RM (2014). PGC-1alpha mediates mitochondrial biogenesis and oxidative phosphorylation in cancer cells to promote metastasis. Nat Cell Biol.

[19] Sancho P, Burgos-Ramos E, Tavera A, Bou Kheir T, Jagust P, Schoenhals M, Barneda D, Sellers K, Campos-Olivas R, Grana O (2015). MYC/PGC-1alpha balance determines the metabolic phenotype and plasticity of pancreatic cancer stem cells. Cell Metab.

[20] Viale A, Pettazzoni P, Lyssiotis CA, Ying H, Sanchez N, Marchesini M, Carugo A, Green T, Seth S, Giuliani V (2014). Oncogene ablation-resistant pancreatic cancer cells depend on mitochondrial function. Nature.

[21] Scandurra FM, Gnaiger E (2010). Cell respiration under hypoxia: facts and artefacts in mitochondrial oxygen kinetics. Adv Exp Med Biol.

[22] Viale A, Draetta GF (2016). Metabolic features of cancer treatment resistance. Recent Results Cancer Res Fortschr der Krebsforsch Progres dans Les Rech sur Le Cancer.

[23] Dando I, Dalla Pozza E, Biondani G, Cordani M, Palmieri M, Donadelli M (2015). The metabolic landscape of cancer stem cells. IUBMB Life.

[24] Palmieri F (2014). Mitochondrial transporters of the SLC25 family and associated diseases: a review. J Inherit Metab Dis.

[25] Palmieri F, Monne M (2016). Discoveries, metabolic roles and diseases of mitochondrial carriers: a review. Biochim Et Biophys Acta.

[26] Kolukula VK, Sahu G, Wellstein A, Rodriguez OC, Preet A, Iacobazzi V, D’Orazi G, Albanese C, Palmieri F, Avantaggiati ML (2014). SLC25A1, or CIC, is a novel transcriptional target of mutant p53 and a negative tumor prognostic marker. Oncotarget.

[27] Catalina-Rodriguez O, Kolukula VK, Tomita Y, Preet A, Palmieri F, Wellstein A, Byers S, Giaccia AJ, Glasgow E, Albanese C (2012). The mitochondrial citrate transporter, CIC, is essential for mitochondrial homeostasis. Oncotarget.

[28] Bielecka ZF, Maliszewska-Olejniczak K, Safir IJ, Szczylik C, Czarnecka AM (2017). Three-dimensional cell culture model utilization in cancer stem cell research. Biol Rev Camb Philos Soc.

[29] Weiswald LB, Bellet D, Dangles-Marie V (2015). Spherical cancer models in tumor biology. Neoplasia.

[30] Sun J, Aluvila S, Kotaria R, Mayor JA, Walters DE, Kaplan RS (2010). Mitochondrial and plasma membrane citrate transporters: discovery of selective inhibitors and application to structure/function analysis. Mol Cell Pharmacol.

[31] Raimundo N, Baysal BE, Shadel GS (2011). Revisiting the TCA cycle: signaling to tumor formation. Trends Mol Med.

[32] Sansbury BE, Jones SP, Riggs DW, Darley-Usmar VM, Hill BG (2011). Bioenergetic function in cardiovascular cells: the importance of the reserve capacity and its biological regulation. Chem Biol Interact.

[33] Nicholls DG (2008). Oxidative stress and energy crises in neuronal dysfunction. Ann N Y Acad Sci.

[34] Li S, Li Q (2014). Cancer stem cells and tumor metastasis (review). Int J Oncol.

[35] Agliano A, Calvo A, Box C (2017). The challenge of targeting cancer stem cells to halt metastasis. Semin Cancer Biol.

[36] Berens EB, Holy JM, Riegel AT, Wellstein A. A cancer cell spheroid assay to assess invasion in a 3D setting. J Vis Exp. 2015. 10.3791/53409. 10.3791/53409 PMC4692745 26649463

[37] Jiang L, Shestov AA, Swain P, Yang C, Parker SJ, Wang QA, Terada LS, Adams ND, McCabe MT, Pietrak B (2016). Reductive carboxylation supports redox homeostasis during anchorage-independent growth. Nature.

[38] Kaplan RS, Morris HP, Coleman PS (1982). Kinetic characteristics of citrate influx and efflux with mitochondria from Morris hepatomas 3924A and 16. Cancer Res.

[39] Trachootham D, Alexandre J, Huang P (2009). Targeting cancer cells by ROS-mediated mechanisms: a radical therapeutic approach?. Nat Rev Drug Discov.

[40] Ciccarese F, Ciminale V (2017). Escaping death: mitochondrial redox homeostasis in cancer cells. Front Oncol.

[41] Birsoy K, Wang T, Chen WW, Freinkman E, Abu-Remaileh M, Sabatini DM (2015). An essential role of the mitochondrial electron transport chain in cell proliferation is to enable aspartate synthesis. Cell.

[42] Sullivan LB, Gui DY, Hosios AM, Bush LN, Freinkman E, Vander Heiden MG (2015). Supporting aspartate biosynthesis is an essential function of respiration in proliferating cells. Cell.

[43] Wu M, Neilson A, Swift AL, Moran R, Tamagnine J, Parslow D, Armistead S, Lemire K, Orrell J, Teich J (2007). Multiparameter metabolic analysis reveals a close link between attenuated mitochondrial bioenergetic function and enhanced glycolysis dependency in human tumor cells. Am J Physiol Cell Physiol.

[44] Hui S, Ghergurovich JM, Morscher RJ, Jang C, Teng X, Lu W, Esparza LA, Reya T, Le Z, Yanxiang Guo J (2017). Glucose feeds the TCA cycle via circulating lactate. Nature.

[45] Newsholme EA, Sugden PH, Williams T (1977). Effect of citrate on the activities of 6-phosphofructokinase from nervous and muscle tissues from different animals and its relationships to the regulation of glycolysis. Biochem J.

[46] Russo A, Franchina T, Ricciardi GR, Picone A, Ferraro G, Zanghi M, Toscano G, Giordano A, Adamo V (2015). A decade of EGFR inhibition in EGFR-mutated non small cell lung cancer (NSCLC): old successes and future perspectives. Oncotarget.

[47] Tan CS, Gilligan D, Pacey S (2015). Treatment approaches for EGFR-inhibitor-resistant patients with non-small-cell lung cancer. Lancet Oncol.

[48] Hirsh V (2018). Turning EGFR mutation-positive non-small-cell lung cancer into a chronic disease: optimal sequential therapy with EGFR tyrosine kinase inhibitors. Ther Adv Med Oncol.

[49] Sullivan I, Planchard D (2016). Osimertinib in the treatment of patients with epidermal growth factor receptor T790M mutation-positive metastatic non-small cell lung cancer: clinical trial evidence and experience. Ther Adv Respir Dis.

[50] Wang T, Narayanaswamy R, Ren H, Torchilin VP (2016). Combination therapy targeting both cancer stem-like cells and bulk tumor cells for improved efficacy of breast cancer treatment. Cancer Biol Ther.

[51] Yuan X, Wu H, Xu H, Han N, Chu Q, Yu S, Chen Y, Wu K (2015). Meta-analysis reveals the correlation of Notch signaling with non-small cell lung cancer progression and prognosis. Sci Rep.

[52] Chen CY, Chen YY, Hsieh MS, Ho CC, Chen KY, Shih JY, Yu CJ (2017). Expression of Notch gene and its impact on survival of patients with resectable non-small cell lung cancer. J Cancer.

[53] Chen Y, Huang Y, Huang Y, Chen J, Wang S, Zhou J (2013). The prognostic value of SOX2 expression in non-small cell lung cancer: a meta-analysis. PLoS ONE.

[54] Svensson RU, Parker SJ, Eichner LJ, Kolar MJ, Wallace M, Brun SN, Lombardo PS, Van Nostrand JL, Hutchins A, Vera L (2016). Inhibition of acetyl-CoA carboxylase suppresses fatty acid synthesis and tumor growth of non-small-cell lung cancer in preclinical models. Nat Med.

