# Supplementary material for: The mitochondrial citrate carrier, SLC25A1, drives stemness and therapy resistance in non-small cell lung cancer
Source: Cell Death Differ. 2018 Apr 12;25(7):1239–58. doi: 10.1038/s41418-018-0101-z (PMC6030199; doi:10.1038/s41418-018-0101-z)
Supplement: Supplementary file 1 — Supplementary Figures and Legends [file 41418_2018_101_MOESM1_ESM.pdf]

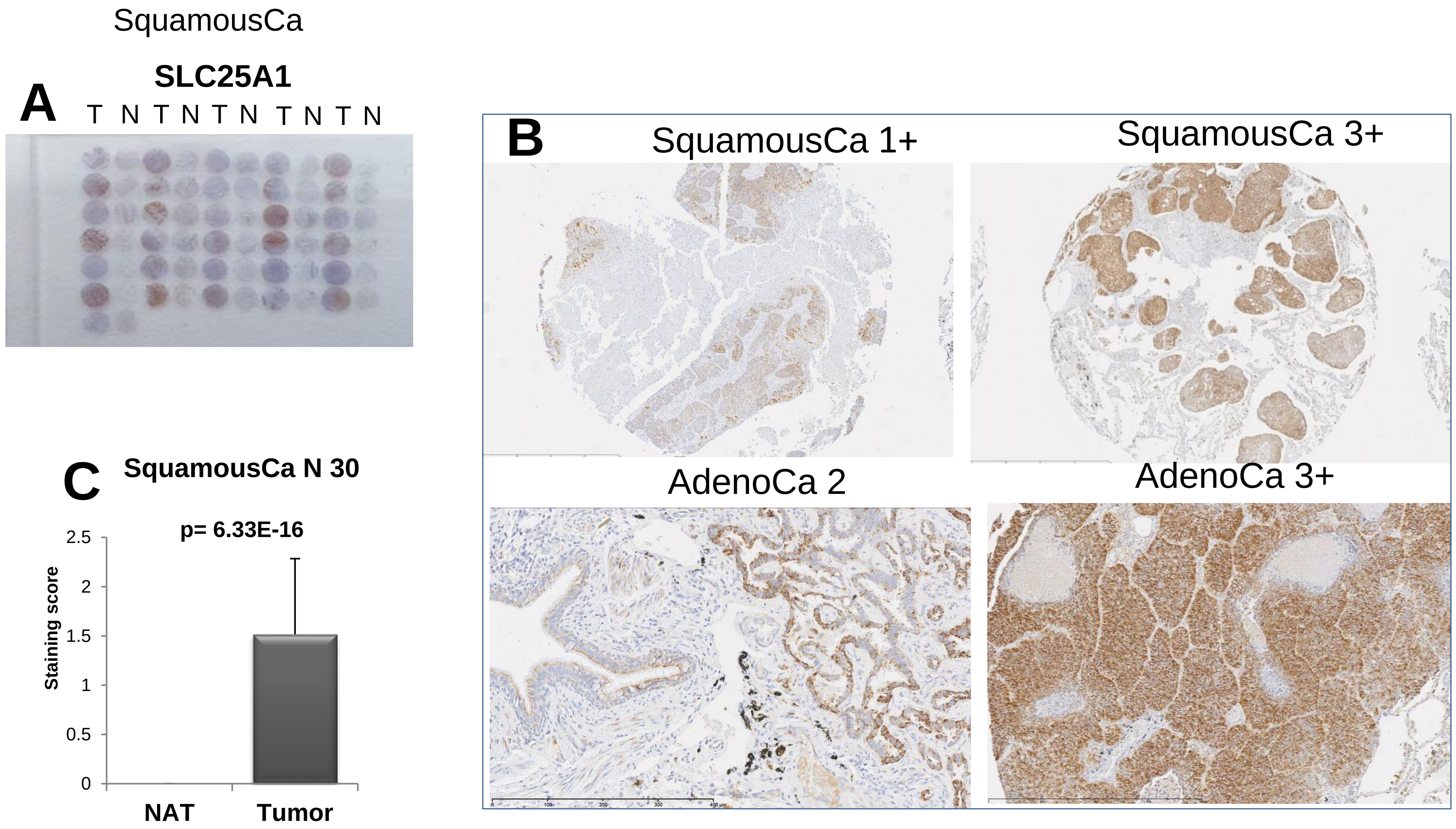

Supplementary Figure 1

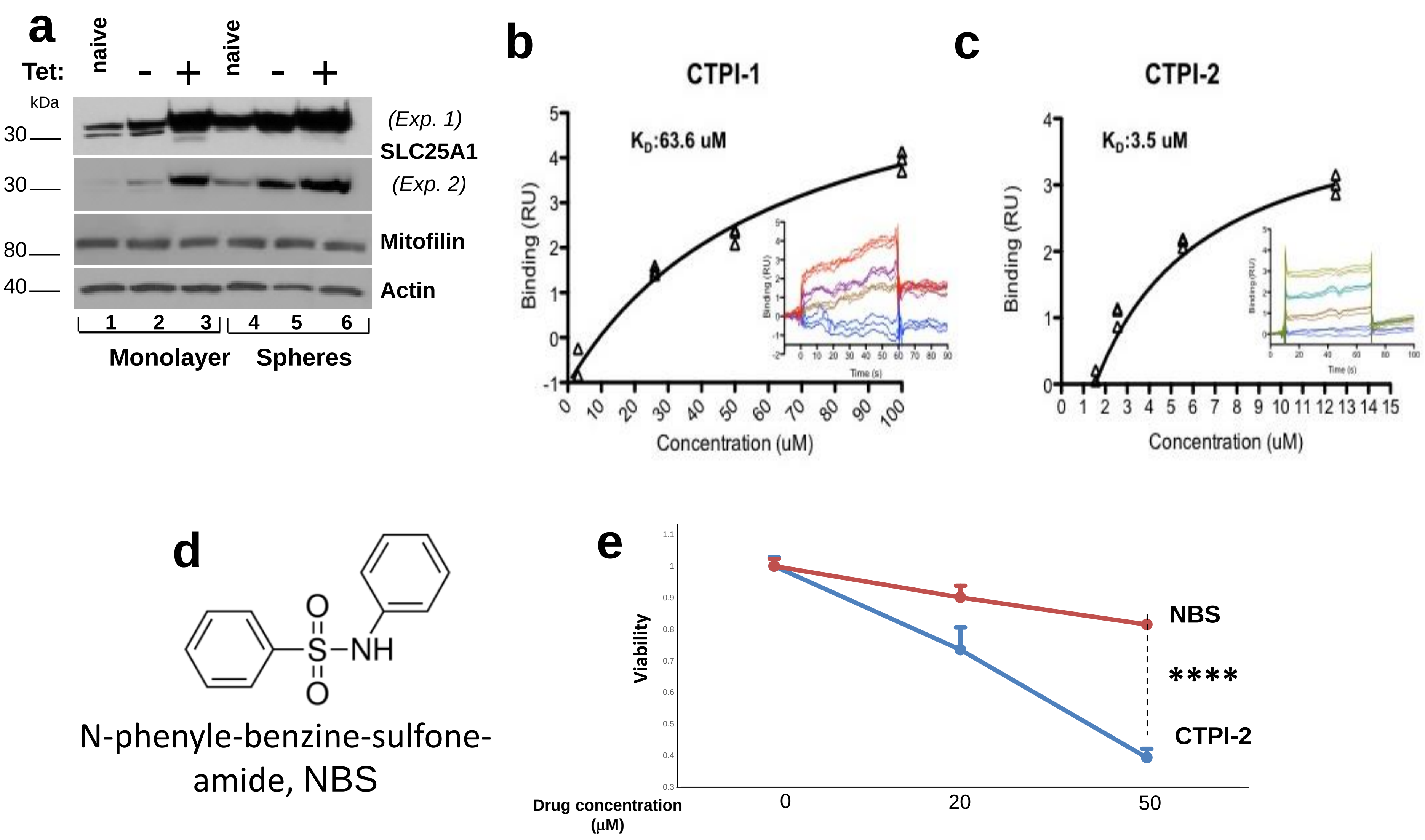

Supplementary Figure 2

Supplementary Figure 3

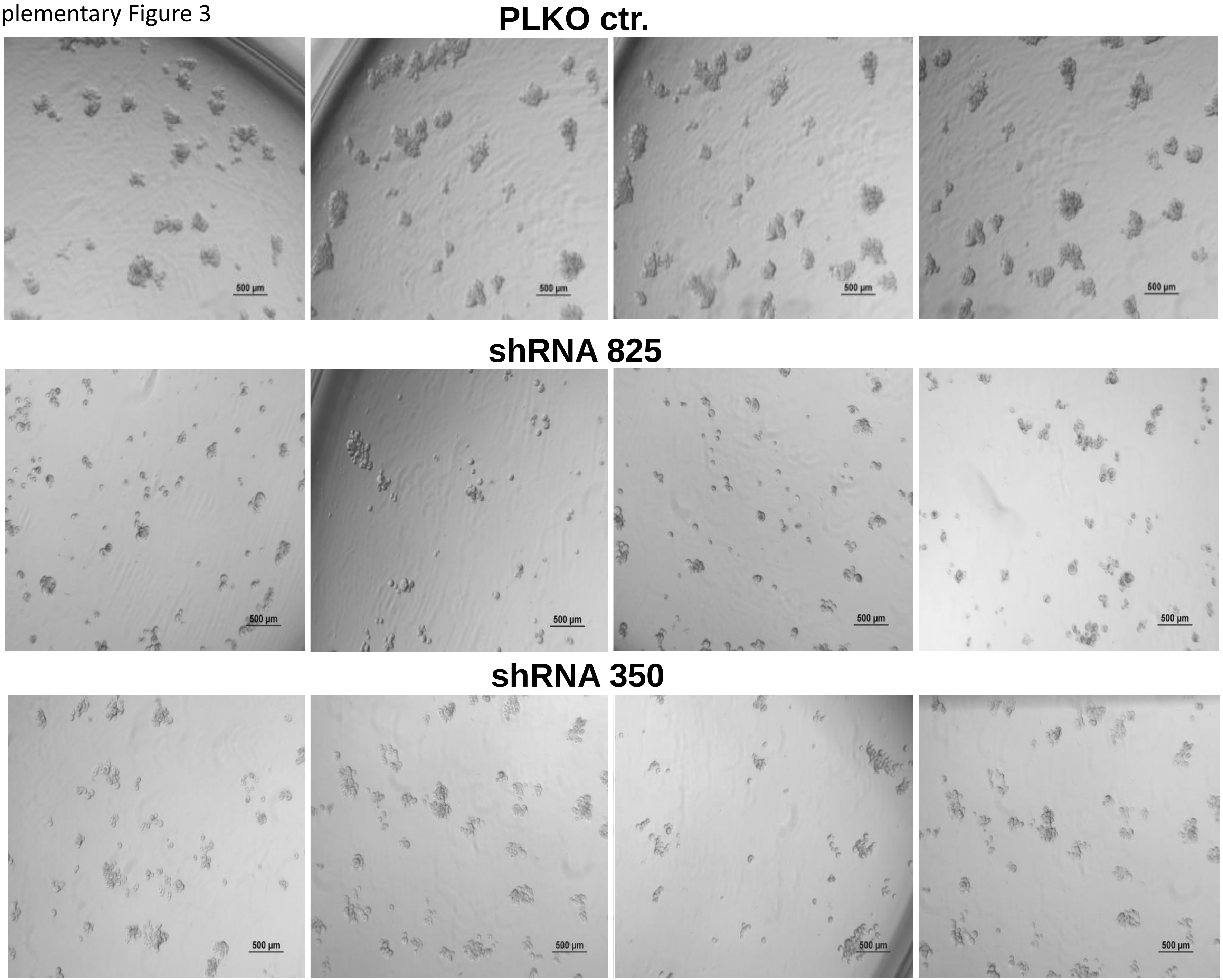

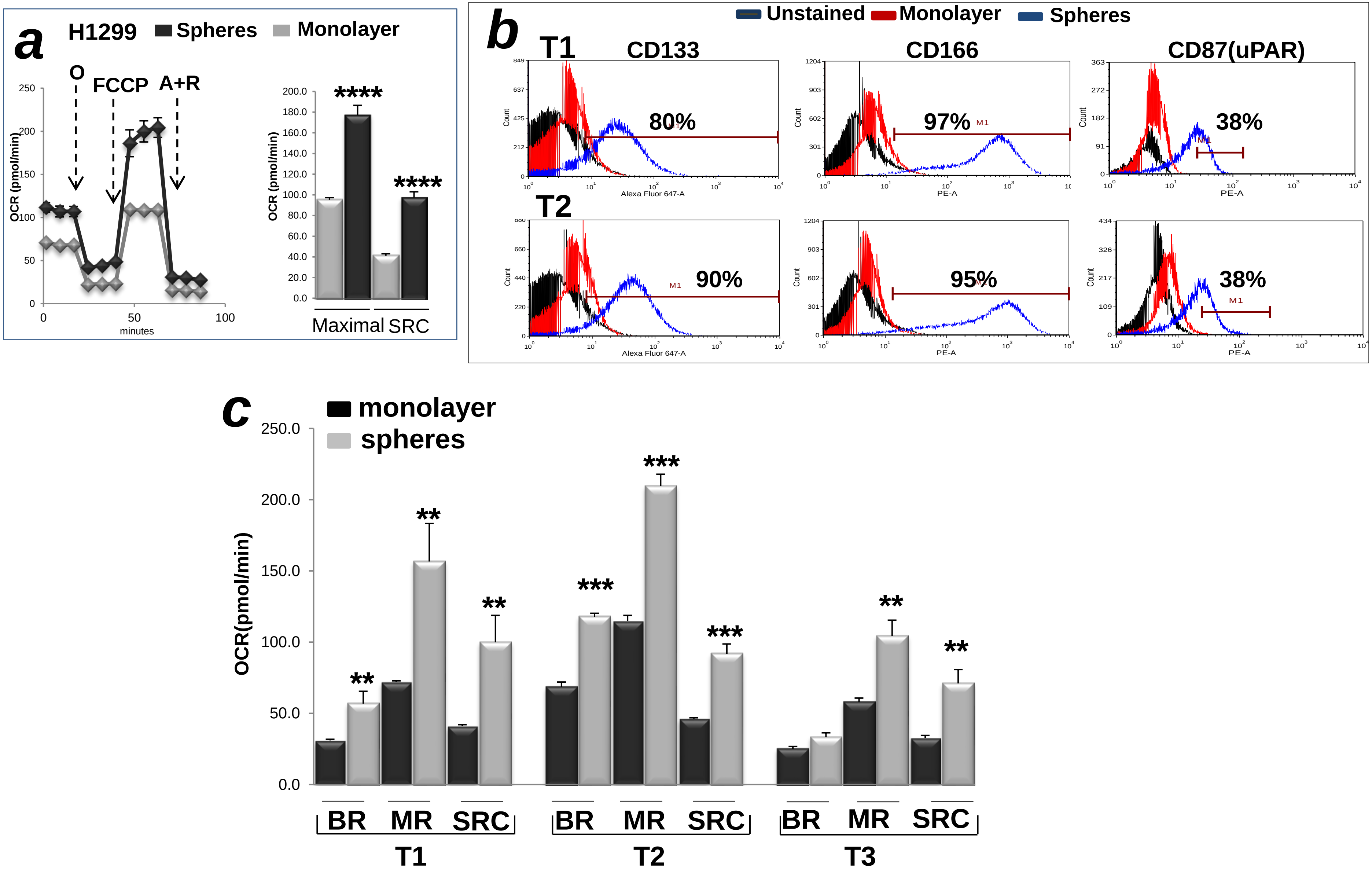

Supplementary Figure 4

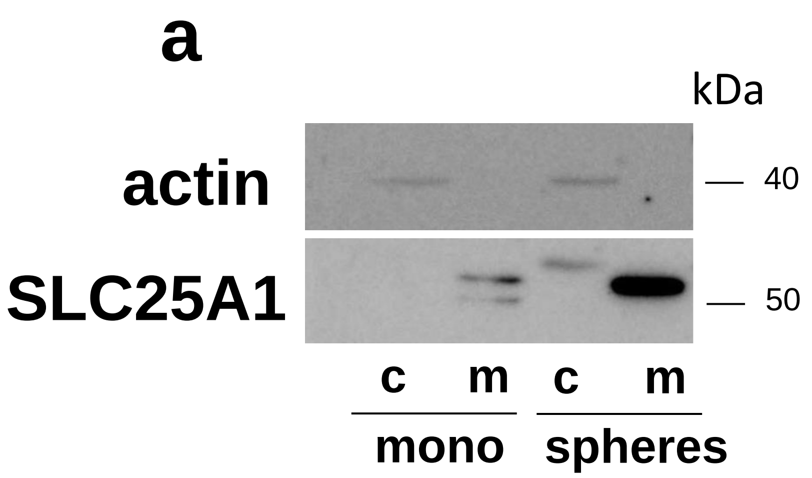

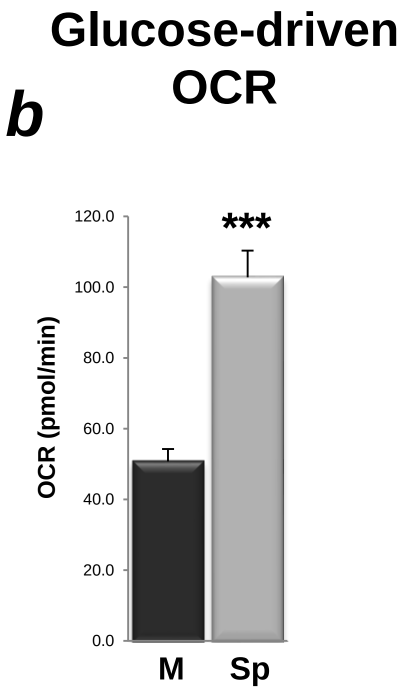

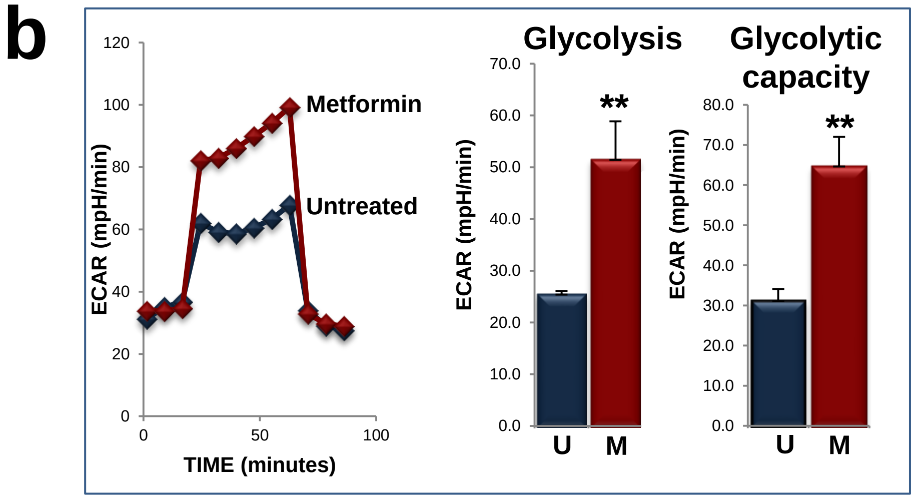

Supplementary Figure 5

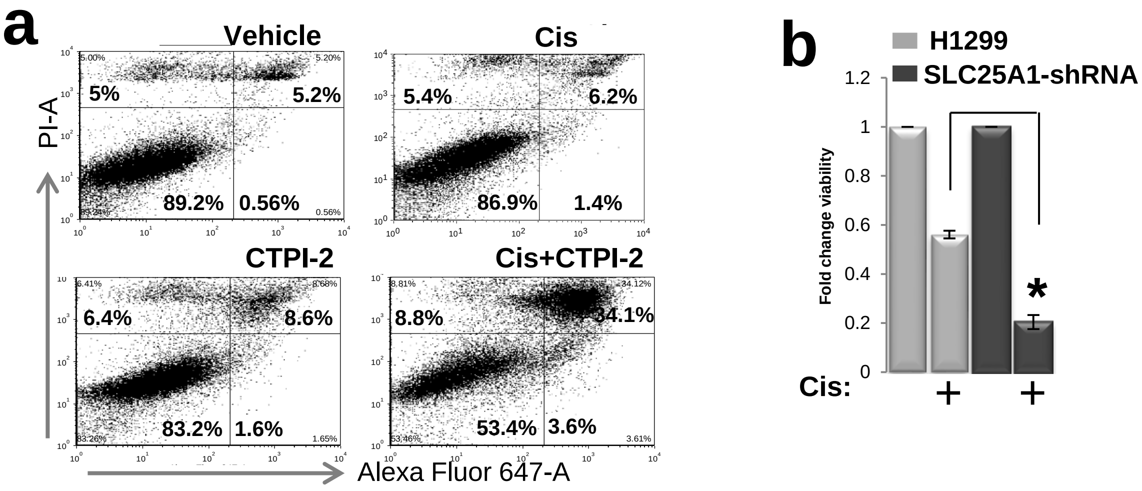

Supplementary Figure 6

Supplementary figure legends

Supplementary Figure 1. Expression of SLC25A1 in tumor arrays. A. Tissue microarrays of 30 matched tumor and normal tissues from squamous carcinoma were stained for SLC25A1. **B**. Scoring criteria examples for Squamous and Adenocarcinomas. **C**. Staining score relative to normal and adjacent normal tissues in squamous carcinoma shows selective positivity in tumors.

Supplementary Figure 2. **Identification and characterization of a novel SLC25A1 inhibitor.** (**a**) Immuno-blots for SLC25A1, mitofilin or actin of H1299 cells harboring tetracycline-inducible SLC25A1 cDNA, grown as monolayer (lanes 1-3), or as spheres (lanes 4-6) in the presence (+) or absence of tetracycline. Two exposures of the SLC251A blot are shown. Note the enrichment of SLC25A1 in spheres versus monolayer cultures (lane 1 versus 4). (**b-c**) Direct binding of CTPI-1 (**a**) and CTPI-2 (**b**) was measured by SPR. Recombinant purified SLC25A1 protein was immobilized on Biacore NTA chip and different concentrations of compounds were injected over the protein coated surface in triplicates (insets show raw data). Binding values from 5 second prior to end of injection were plotted against compound concentration to calculate steady state binding affinity (K_D) based on a 1:1 binding model in BiaEvaluation software. (**d**) Structure of the backbone scaffold compound, N-phenyle-benzine-sulfone-amide (NBS) lacking the groups necessary for interaction with CTPI-2 (see also text for explanation). (**e**) Growth curves of cells treated with NBS or CTPI-2.

Supplementary Figure 3. Representative images of cells infected with lentivirus Plko or with the SLC25A1 shRNA, related to quantification experiments shown in Figure 2h.

Supplementary Figure 4. Sphere cultures enriched in CSCs show enhanced levels of OCR. (**a**) Oxygen consumption rates (OCR), maximal and spare respiratory capacity (SPR) were assessed using the Seahorse Extracellular Flux Analyzer after injection of oligomycin, FCCP and Antimycin/Rotenone in H1299 cells grown as monolayer or spheres. (**b**) Analysis with FACS of the CD166, CD133 and CD87 markers in the T1 and T2 cells grown as monolayer or spheres, demonstrating the enrichment (near 100%) of stem cell populations. (**c**) Normalized OCR levels in T1, T2 and T3 cells grown as monolayer or CSCs-spheres. Once spheres were formed cells were dissociated, plated in 96 wells and incubated in the same media for 6-12 before assessment of OCR. Treatment with CTPI-2 was for 3 hours.

Supplementary Figure 5. Differential effects of metformin and CTPI-2 on glycolysis. (**a**) Mitochondrial (m) and cytoplasmic (c) extracts were probed in immune-blot with the indicated antibodies. (**b**) Levels of glucose-driven OCR, or glucose oxidation, extrapolated after injection of glucose and then of the glycolytic inhibitor 2-deoxyglucose by using the extracellular seahorse analyzer. (**c**) Dynamic

profile and levels of basal and glycolytic capacity in cells treated with metformin assessed with the Seahorse extracellular flux analyzer.

Supplementary Figure 6. (**a**) Flow cytometry analysis with annexin V and PI staining of T1 cells treated with Cisplatin or CTPI-2, alone or in combination. (**b**) H1299 cells mock-infected or infected with the SLC25A1 lentivirus were established for one week after selection in puromycin. Cells were treated with cisplatin (5 □M) and viability was assessed with trypan blue exclusion after five days.
